# Integrating features of radiomics and CNN models for early skin cancer detection based on watershed segmentation

**DOI:** 10.1007/s12672-026-04652-7

**Published:** 2026-02-18

**Authors:** Abdullah Shoaib Alshmrani, Fahad M. Alotaibi, Ahmed S. Alfakeeh

**Affiliations:** Department of Information Systems, Faculty of Computing and Information Technology (FCIT), King Abdulaziz University, Jeddah, 21589 Saudi Arabia

**Keywords:** CNN, Radiomic, Watershed, ANN, RF, Combining features, Skin cancer

## Abstract

Skin cancer is among the most aggressive and prevalent forms of cancer worldwide, with melanoma posing a high risk of metastasis and mortality when not detected early. Manual diagnosis by dermatologists, while effective, faces challenges such as subjectivity, variability, and limited accessibility in underserved regions. To address these limitations, this study proposes a robust computer-aided diagnostic system for early detection of skin cancer using a hybrid feature extraction approach and machine learning. In this study, several methodologies were developed for the automated classification of dermoscopic images, with a primary focus on a hybrid diagnostic model combining radiomic and deep learning features. Specifically, a Random Forest (RF) classifier was trained on fused features extracted from radiomic algorithms and deep convolutional layers of CNN. The proposed MobileNetV2 + radiomics + RF model achieved outstanding performance, with an average AUC of 85.09%, accuracy of 94.7%, sensitivity of 84.53% and specificity exceeding 99.2%. It exhibited particularly strong classification capabilities for high-risk lesions such as melanoma (AUC of 94.4%) and nevi (AUC of 98.2%), while maintaining robust performance across other lesion types. The integration of radiomic and CNN-based features through an RF classifier offers a highly effective approach for early skin cancer detection, with significant implications for clinical practice.

## Introduction

 The skin is the largest organ of the human body and constitutes the first line of defense against pathogens [1], UV light, and physical assault [2]. It helps control temperature, prevents dehydration, and enhances the sense of touch. Benign and malignant conditions are included in the classification of skin lesions (nevi, seborrheic keratosis) [3]. Severe lesions are melanoma (MEL), basal cell carcinoma (BCC), and squamous cell carcinoma (SCC) [4]. Melanoma, originating from melanocytes, is the deadliest due to its high potential for lesions and mortality rate if not detected early [5]. Manual diagnosis is prone to several limitations that render it susceptible to errors. Doctors’ opinions differ, especially in the early stages [6]. Melanomas present with unclear characteristics in the early stages, leading to misclassification as benign moles or other lesions [7]. The scarcity of skilled doctors relative to the number of patients, especially in remote areas, also poses a challenge [8]. AI technologies, particularly deep CNNs, have emerged as transformative tools for skin lesion analysis [9]. Their ability to learn hierarchical feature representations directly from data allows them to recognize subtle and complex patterns that may elude the human eye [10]. This capability helps reduce diagnostic subjectivity, standardize procedures, and expand access to screening [11]. However, the performance of CNNs is intrinsically linked to their architectural biases—such as receptive field size and layer depth—which guide the types of features they learn to prioritize [12].

A critical observation in the field is that while CNNs excel at learning complex data-driven features, they may not fully encapsulate the clinically meaningful [13], hand-crafted descriptors—such as specific texture metrics, color variegation, and border irregularity—that dermatologists routinely use for diagnosis [14]. This creates a methodological gap. Many state-of-the-art approaches refine CNN architectures or data handling but remain within a single feature paradigm [15] —either deep learning or manual —and neglect their potential synergy [16]. For instance, some studies achieve high accuracy using ensembles of CNNs [17], while others focus on advanced segmentation [18] or imbalance mitigation [19, 20], yet they do not integrate handcrafted radiomic features [21]. Consequently, these models might struggle with lesions where clinically established visual cues are paramount but not fully captured by the learned filters. Deep features derived from a single CNN may excel at highlighting pigment variation across a lesion [19]. Once the composite vector is generated, it is fed into a set of traditional machine learning classifiers. Each classifier is trained on a labeled training set of skin images, using five-way cross-validation to select hyperparameters that improve classification accuracy, sensitivity, and specificity [22]. In this way, the combined representation utilizes the fine-grained abstractions learned from multiple CNN architectures, along with manual features used by dermatologists in clinical practice [23].

This study addresses this gap by proposing a hybrid framework that systematically integrates handcrafted radiomic features with deep learning representations. Our core contribution is the creation of a unified feature vector that combines the strengths of both approaches: the quantitative power of radiomics (texture, spatial relationships, color) and the hierarchical abstraction power of deep features from multiple CNN architectures. We posit that this fusion creates a more comprehensive and discriminative representation of each lesion.

The major contributions of this work are summarized as follows:

- Dermoscopic images were preprocessed to enhance clarity by applying mean filtering and automated hair removal, ensuring cleaner lesion visibility and more accurate feature extraction.
- Four radiomic feature extraction techniques—Local Binary Patterns (LBP), Gray Level Dependence Matrix (GLDM), Gray Level Size Zone Matrix (GLSZM), and Color Histograms—were employed and combined to form a comprehensive radiomic representation.
- A novel hybrid feature vector was engineered by concatenating the handcrafted radiomic features with deep features extracted from pre-trained MobileNetV2 and EfficientNet-B4 models.
- Three machine learning classifiers—ANN, SVM, and RF—were rigorously evaluated. The Random Forest classifier applied to the hybrid features, particularly from MobileNetV2, demonstrated superior performance, validating the effectiveness of our fusion strategy.

The study is organized as follows: Sect.  2 reviews related studies. Section  3 details the preprocessing, feature extraction, and classification methodologies. Section  4 presents and analyzes the experimental results. Section  5 provides a comparative discussion. Finally, Sect.  6 concludes the study.

## Related work

Numerous studies have sought to improve the automatic analysis of dermoscopic images by leveraging CNN architectures; yet, many remain limited by a narrow focus or an insufficient evaluation of complementary techniques. For instance, Toprak et al. [24] proposed a hybrid strategy that combines DeepLabV3 + for lesion segmentation with multiple CNNs (MobileNetV2, EfficientNetB0, and DenseNet201) for feature extraction. Although their reported accuracies on ISIC 2019 and PH2 (94.42% and 94.44%, respectively) exceed those of single CNNs, their method relies solely on learned features. It does not investigate how hand-crafted descriptors might complement these deep representations. Houssein et al. [25] introduced a CNN architecture optimized explicitly for class-imbalanced data, integrating transfer learning with in-network feature optimization. Evaluated on HAM10000 and ISIC-2019, this model demonstrated improved multi-metric performance, yet its design does not explicitly incorporate domain-specific descriptors (asymmetry or color distribution measures). Consequently, although it mitigates imbalance, the model may overlook meaningful cues that are difficult for convolutional filters alone to capture, especially in cases of low-contrast or unevenly pigmented lesions. Similarly, Nigar et al. [20] achieved notable eight‐class classification accuracy (94.47%) and recal (94.01%) on ISIC 019 using a CNN model. However, their work does not evaluate the contribution of classical, hand-crafted features, such as texture metrics or shape indices, that dermatologists routinely use in clinical practice. Hosny et al. [26] explored residual CNNs for melanoma detection and tested segmented versus unsegmented approaches across PH2, ISIC 2017, and ISIC 2018. Although they report improved generalization when training on cross‐dataset images, their analysis stops short of assessing how explicitly engineered features (e.g., color asymmetry or border sharpness) could augment CNN-learned representations. Other works have focused on preprocessing or architectural refinements. Gouda et al. [18], for example, applied ERGAS-based image enhancement before feeding images into a transfer‐learned CNN, achieving 83.2% accuracy on ISIC 018. While their enhancement step improved overall image quality, no explicit quantitative comparison shows how hand-crafted texture or color features might complement the enhanced deep features. Singh et al. [27] integrated neutrosophic thresholding segmentation and novel histogram equalization before CNN classification, reporting strong performance on PH2, ISIC 2018, and ISIC 2019. Yet their pipeline remains entirely CNN‐centric in feature representation, thereby forgoing potentially complementary descriptors. Several authors have addressed class imbalance and data augmentation. Gayatri et al. [28] applied focal loss with ResNet50 to reduce overfitting on difficult classes, and Nawaz et al. [29] used extensive augmentation before training MobileNetV3 and EfficientNetV2B0. Although these strategies improve class balance, they still treat CNN outputs as sole descriptors, neglecting the explicit incorporation of hand‐engineered features that might capture lesion attributes not readily learned through convolutional filters. Others have emphasized segmentation accuracy. Ahmed et al. [30] combined RetinaNet with Mask R‐CNN for precise lesion delineation. Yet, they do not explore how segmentation masks can guide the extraction of shape-based or color-based metrics for downstream classification. Amin et al. [31] employed a U–Net–based model with a hybrid loss to achieve high IoU and Dice scores for lesion segmentation; however, no subsequent analysis has evaluated how combining classical feature metrics with CNN outputs might enhance final classification tasks. CNN variants, such as EfficientNet (Harahap et al. [32]) and stacked architectures (Mui-zzud-din et al. [33], Khan et al. [34]), have demonstrated accuracy improvements—often in the low-90% range—on the HAM10000 and ISIC datasets.

Still, these studies share a standard limitation: they focus on refining convolutional blocks, loss functions, or data-handling strategies, without adequately considering how traditional hand-crafted features (color, texture measures, asymmetry indices) could complement CNN representations and address scenarios where learned filters alone may struggle (as, in lesions with subtle border irregularities or low‐contrast color variation). This work builds upon these strategies by integrating CNN and machine learning models, enhancing lesion segmentation, and adopting hybrid learning models to improve generalization and real-world diagnostic accuracy using the ISIC 2019 dataset.

## Materials and tools

**Fig. 1:**
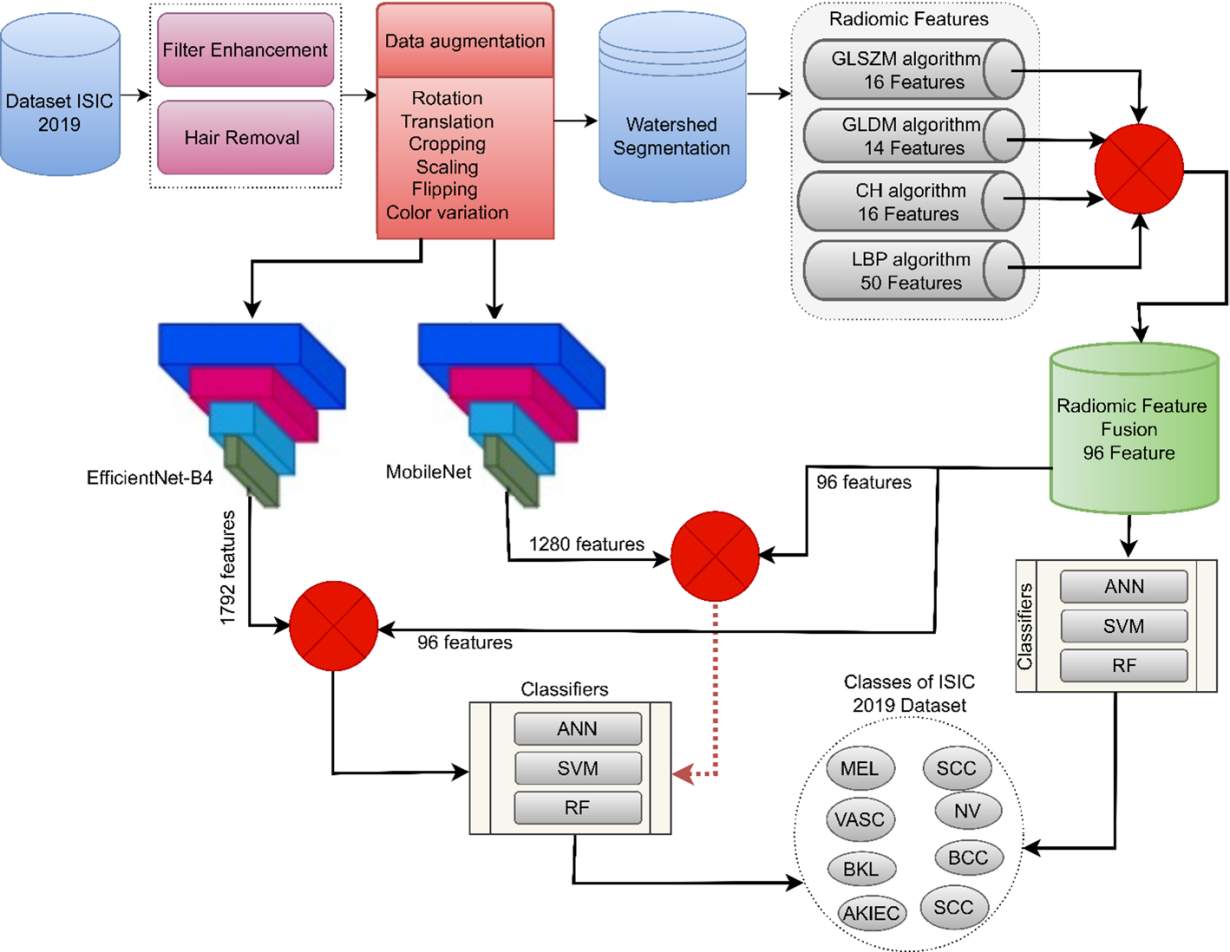
Framework for a methodology for analyzing dermoscopy images for skin cancer detection

Figure 1 illustrates the proposed pipeline for skin lesion classification using the ISIC 2019 dataset. Initially, dermoscopic images undergo enhancement through filtering and hair removal to ensure cleaner lesion visualization. Data augmentation is then applied, including transformations such as rotation, scaling, cropping, and flipping to improve generalization. Following this, watershed segmentation is employed to isolate the lesion. From the segmented lesions, radiomic features are extracted using four algorithms: GLSZM (16 features), GLDM (14), CH (16), and LBP (50), resulting in a 96-feature fused vector. These features are then classified using three machine learning models—ANN, SVM, and RF. Deep features were extracted using the MobileNetV2 and EfficientNet-B4 models, combined with radiomics features, and classified using ANN, SVM, and RF classifiers [35].

### Description of dermoscopy ISIC-2019 datasets

The ISIC-2019 dataset aggregates 25,331 high-resolution dermoscopic images sourced from leading tertiary care centers, acquired using diverse dermoscopy devices (e.g., Heine, Canfield). The proposed systems employ a 5-fold cross-validation strategy to ensure robust model generalization and prevent overfitting. Specifically, 80% of the ISIC-2019 dataset was allocated for training and validation, while the remaining 20% was used for independent testing. Within each fold, 80% of the training subset was used for model training and 20% for validation. This procedure ensured balanced evaluation across all classes, including minority categories, as summarized in Table 1, thereby strengthening the statistical reliability of the reported results. There is a notable class imbalance, with Nevi (NV) having 12,875 images compared to just 253 images for Vascular (VASC), which presents a challenge for systems that typically prioritize the majority classes. Nevertheless, this challenge has been addressed through the use of data augmentation techniques [36].

**Table 1:** Dividing the dermoscopy images from ISIC 2019 related to skin lesions

Classes	Training (64%)	validation (16%)	Testing 20%
Actinic Keratoses	555	139	173
Basal cell carcinoma	2126	532	665
Benign Keratosis Lesions	1679	420	525
Dermatofibroma	153	38	48
Melanoma	2894	724	904
Nevi	8240	2060	2575
Squamous cell carcinoma	402	100	126
Vascular	162	40	51

### Preprocessing

Preprocessing is the initial step in our image analysis pipeline. It aims to reduce noise, enhance essential structures, and remove artifacts (such as hairs) that can interfere with subsequent skin-lesion segmentation and feature extraction. In this study, we apply a combination of smoothing and sharpening filters—namely, the average filter and the Laplacian filter—to improve image clarity [37]. The DullRazor technique is employed to detect and eliminate hairs from each dermoscopic image. Details of each substage are given below.

#### Laplacian and average-filter enhancement

Image enhancement restores and accentuates features that may be obscured by noise, uneven illumination, or low contrast between the lesion and the surrounding skin. We proceed in two main steps [38]:

Average Filter:

Begin by convolving each image with a 5 × 5 Average filter. In practice, this filter moves a 5 × 5 window across the image and replaces the value of the central pixel.

z (i, j) with the arithmetic mean of its M is 25 neighbors. Formally, if *y (i*,* j)* denotes the original pixel values, then after applying the Average filter as in Eq. 1.

1 $$I_{{avg}} (i,\:j) = \frac{1}{{m\: \times \:\:n}}{\sum\limits_{{a = - a}}^{a} {\sum\limits_{{n = - b}}^{b} {{{\bar{\mathrm{I}}}}({\text{i + m,}}\,{\text{j + n}})} } } $$

Where: $\:{\hspace{0.05em}}\stackrel{\prime }{I}\left(i,\:j\right)\:$intensity of the input image at pixel (i, j), $\:{I}_{avg}(i,\:j)$ intensity of the image after applying the Average filter, $\:m\:x\:n$ size of the filter window and the summation run over the neighborhood centred at (*i*,* j*).

Laplacian Filter:

Next, the Laplacian operator is applied to the image to emphasize regions of rapid intensity change (edges) [39]. The continuous Laplacian of the image function *f (x*,* y*) is given by Eq. 2 [40].

2 $$\:{\nabla\:\:}^{2}\:f=\frac{{\partial\:\:}^{2}\:f}{{\partial\:}^{\:2}\:x}+\:\frac{{\partial\:\:}^{2}\:f}{{\partial\:}^{\:2}\:y}$$

The 3 × 3 Laplacian kernel is used to approximate these second derivatives. When convolved with the image, the Laplacian output highlights lesion boundaries and fine structural details.

Combining Average and Laplacian Outputs:

Finally, merge the Laplacian-enhanced image with the mean-enhanced image to obtain the sharpened result.

To mitigate the impact of dark hair on subsequent image analysis stages, a standardized hair removal preprocessing step was applied to dermoscopy images [41]. We used the well-known DullRazor technique [41–44] to address this common issue [42]. This method improves data accuracy by detecting and digitally recoloring hair pixels, reducing potential interference with lesion segmentation and feature extraction algorithms [43]. Applying this technique ensures that the extracted quantitative features and segmentation are more representative of the lesion’s shape and texture, rather than being distorted, thereby enhancing the robustness of the subsequent diagnostic model [44]. The specific algorithmic details for morphological closure of hair detection and bilinear interpolation of recoloring follow the conventional implementation described in the literature.

### Data augmentation

Data augmentation artificially increases training data diversity by applying label-preserving transformations to existing images, thereby helping to balance class distributions, prevent overfitting, and improve model robustness and generalizability. We applied the following augmentation operations: geometric transforms, including horizontal and vertical flipping, rotation (± 15°), and zooming—colour transformations: brightness, contrast, and saturation variation. To achieve approximate balance, we set a target number of ~ 8000 images per class (based on the majority class “Nv”) and generated augmented samples for minority classes accordingly [45].

Crucially, to prevent any data leakage, the dataset was first split into training, validation, and test sets. Data augmentation techniques were applied exclusively to the training set images after the split. The validation and test sets remained completely untouched by any augmentation, ensuring an unbiased evaluation of the model’s performance on original, real-world data.

**Fig. 2:**
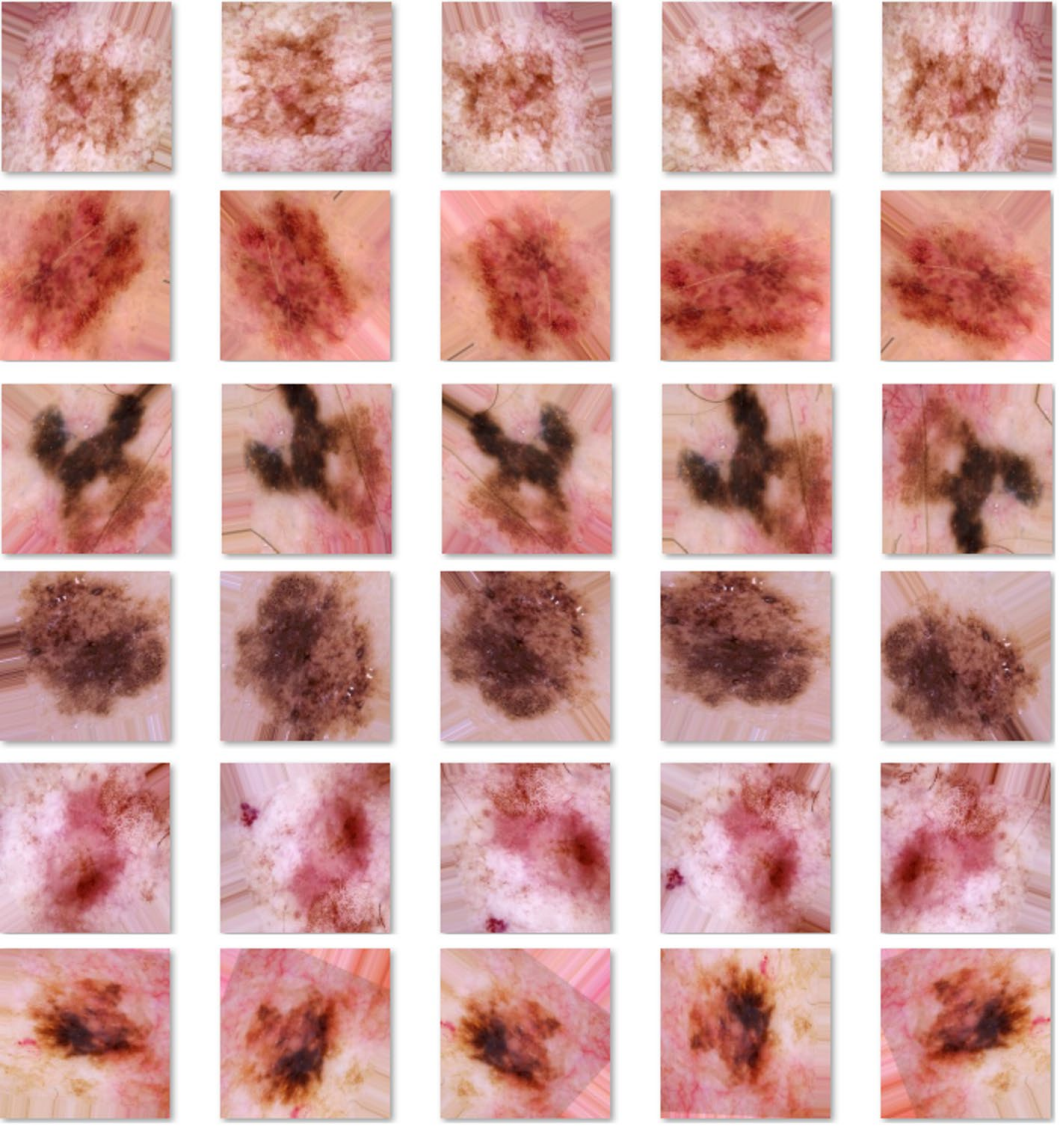
Dermoscopic images from the original and artificially generated ISIC 2019 dataset

To address the severe class imbalance in the ISIC 2019 dataset, we implemented class-specific augmentation strategies. The number of augmentation repetitions varied depending on the initial number of images per class. Specifically, the Dermatofibroma (DF) and Vascular categories, having the smallest representation, were expanded by generating 51 synthetic variants of each image [46]. The Actinic Keratoses Intraepithelial Carcinoma (AKIEC) images were similarly expanded 13 times, while SCC images were enhanced 19 times. The BCC and benign keratosis-like lesions (Bkl) categories were augmented 3 and 4 times, respectively [47]. For MEL cases, the dataset was doubled in size. Importantly, NV images were left unaltered due to their dominance in the dataset, rendering further augmentation unnecessary [48]. his strategic augmentation resulted in balanced training sets, ranging from 7,770 to 8,682 images per category, with each class reaching approximate parity with the NV reference, while preserving diagnostic features through carefully constrained transformations, as shown in Table 2. The geometric and photometric modifications (flipping, rotation, color variation) were implemented such that all synthetic images maintained clinical validity and label integrity. The differential augmentation approach effectively addressed both the quantitative insufficiency of rare classes and the qualitative imbalance that could bias classifier training. By tailoring the augmentation intensity to each class’s original prevalence, the method created a more equitable training environment while maximizing the utility of all available clinical samples. This methodology is visually illustrated in Fig. 2, which demonstrates the transformation from highly skewed original distributions to properly balanced augmented sets suitable for robust model development.

**Table 2:** Allocation of images among ISIC2019 categories before and following data augmentation

Phase	Training dataset							
Classes	Scc	Akiec	Bcc	Bkl	Df	Mel	Nv	Vasc
Before augmentation	402	555	2126	1679	153	2894	8240	162
After augmentation	**8040**	**7**,**770**	**8**,**504**	**8**,**395**	**7956**	**8**,**682**	**8240**	**8424**

### Watershed segmentation

Watershed segmentation treats a grayscale image as if it were a topographic surface, where pixel intensities correspond to “elevation” values. High-gradient regions (sharp intensity changes) form ridges, while homogeneous areas correspond to basins. By imagining that water gradually floods this relief from its minima, the boundaries where water from different basins meet define the “watershed lines,” which serve as segmentation contours. In our implementation for ISIC-2019 dermoscopic images, the goal is to separate lesion regions (diseased skin) from surrounding healthy skin [49].

Gradient-Magnitude Computation:

First, the original color image *I*_*RGB*_ *(x*,* y)* is converted to grayscale *I (x*,* y)*. The gradient magnitude ∣∇I (x, y)∣ is then computed using a Sobel operator in the horizontal and vertical directions as Eqs. 3–5 [50].

3 $$\:{G}_{x}\left(\mathrm{x},\:\mathrm{y}\right)=I\left(x+1,y\right)-I\left(x-1,y\right)\:$$

4 $$\:{G}_{y}\left(\mathrm{x},\:\mathrm{y}\right)=I\left(x,y+1\right)-I\left(x,y-1\right)\:$$

5 $$\:\mid\:\nabla\:I\:\left(\mathrm{x},\:\mathrm{y}\right)\mid\:=\sqrt{{{G}_{x}\left(\mathrm{x},\:\mathrm{y}\right)}^{2}+{{G}_{y}\left(\mathrm{x},\:\mathrm{y}\right)}^{2}}\:\:\:$$

The resulting gradient image ∣∇I∣ highlights the edges of lesions, because lesion borders produce high-magnitude values.

Marker Extraction (Foreground and Background):

Watershed performs best when supplied with markers indicating at least one “definite foreground” region (lesion) and one “definite background” region (healthy skin). The derive these markers as follows:

Sure Background (B_sure_​) [51].

Apply an adaptive threshold T_bg_​ on the smoothed image to identify large homogeneous background areas as Eq. 6:

6 $$\:{B}_{sure}\left(\mathrm{x},\:\mathrm{y}\right)=\:\left\{\begin{array}{c}1\:\:\:\:\:\:\:\:\:\:if\:{I}_{med}\left(\mathrm{x},\:\mathrm{y}\right)\:>\:{T}_{bg},\:\\\:0\:\:\:\:\:\:\:\:\:\:\:\:\:\:\:\:\:\:\:\:\:\:\:\:\:\:\:otherwise\end{array}\right.$$

After thresholding, perform one iteration of morphological closing with a 5 × 5 structuring element to fill small holes in the background.

Sure Foreground (F_sure_):

Compute the Euclidean distance transform D (x, y) on the binary inversion of B_sure_​. Let D denote the distance map as Eq. 7:

7 $$\:\mathrm{D}\:\left(\mathrm{x},\:\mathrm{y}\right)={min}_{\left(u,v\right)\in\:\text{}}\sqrt{{\left(\mathrm{x}-\:\mathrm{u}\right)}^{2}+{\left(\mathrm{y}-\:\mathrm{v}\right)}^{2}}\:\:\:\:\:\:\:\:\mathrm{S}=\left\{\right(\mathrm{u},\:\mathrm{v})\mid\:{B}_{sure}\left(\mathrm{x},\:\mathrm{y}\right)=0.$$

Then threshold D (x, y) at a fraction τ of its maximum value to obtain the sure-foreground mask as Eq. 8:

8 $$ F_{{sure}} \left( {{\mathrm{x}},\:{\mathrm{y}}} \right) = \:\left\{ {\left\{ {\begin{array}{*{20}c} {1~~~~~~~~~~if~D\left( {{\mathrm{x}},{\mathrm{~y}}} \right)~ > ~\tau ~max\left( {D\left( {{\mathrm{x}},{\mathrm{~y}}} \right)} \right),~} \\ {0~~~~~~~~~~~~~~~~~~~~~~~~~~~~~~~~~~~~~~~~~~~~otherwise} \\ \end{array} } \right.} \right.\:\: $$

Marker Labeling:

Next, each connected component in F_sure_​ is assigned a unique integer label as Eq. 9.

9 $$ $$$$$$$$ M~\left( {{\mathrm{x}},{\mathrm{~y}}} \right) = \:\left\{ {\begin{array}{*{20}c} 0 & {~if~B_{{sure}} \left( {{\mathrm{x}},{\mathrm{~y}}} \right) = 1,} \\ {k~{\mathrm{if~Bsure}}\left( {{\mathrm{x}},{\mathrm{y}}} \right) = 1,} & {{\mathrm{if~pixel~}}\left( {{\mathrm{x}},{\mathrm{y}}} \right){\mathrm{~belongs~to~the~k}} - {\mathrm{th~connected~component~of~}}F_{{sure}} } \\ \end{array} } \right.\:\:\: $$

where, *M (x*,* y)* = 0 marks the background region and positive integers *k* mark individual lesion-foreground markers. $\:{k}_{th}\:$refers to the unique, connected region identified within the sure-foreground mask, $\:{F}_{sure}$.

Applying the Watershed Transform:

The classical watershed transforms flood the gradient image ∣∇*I*∣ from the labeled markers *M (x*,* y).* At each iteration, pixels of lowest gradient magnitude adjacent to already flooded regions are incorporated into the corresponding basin [52]. When two basins attempt to flood the same pixel, a watershed line is placed at that boundary. The resulting label map $\:L\:\left(\mathrm{x},\:\mathrm{y}\right)$ satisfies as Eq. 10:

10 $$ \:L\:\left( {{\mathrm{x}},\:{\mathrm{y}}} \right) = \left\{ {\begin{array}{*{20}c} {i\:\:\:\:\:\:\:\:\:\:\:\:\:\:\:\:\:\:\:\:\:\:\:\:\:\:\:\:\:\:\:\:\:\:\:\:\:\:\:\:\:\:\:\:\:\:\:\:\:\:\:\:\:\:\:\:\:\:\:if\:pixel\:(x,y)\:belongs\:to\:basin\:i,} \\ {\:0\:\:\:\:if\:pixel\:(x,y)\:is\:assigned\:to\:a\:watershed\:ridge\:\left( {boundary} \right).} \\ \end{array} } \right.\: $$

Each positive integer *i* indicates a segmented region; typically, the largest basin (or the basin with largest overlap with $\:{F}_{sure}$ is selected as the lesion mask.

Post-Processing and ROI Extraction:

Once $\:L\:\left(\mathrm{x},\:\mathrm{y}\right)$ is computed, regions labeled I > 0 are examined. The label corresponding to the largest intersection with $\:{F}_{sure}$​ is retained as the final lesion mask $\:{L}_{lesion}$(x, y). Any small spurious basins—those whose area falls below a threshold $\:{\alpha\:}_{min}$ ​—are discarded. Finally, the bounding box of $\:{L}_{lesion}$ is used to crop the original RGB image, yielding a Region of Interest (ROI) that contains only the diseased area. These ROIs are saved to a separate folder for downstream feature extraction and classification.

Several methodological considerations drove the selection of the marker-controlled Watershed algorithm. Its primary advantage lies in its model-free, unsupervised nature, which eliminates the need for a large dataset of pixel-level annotations required to train a supervised model like U-Net. This makes it highly data-efficient and reproducible. Furthermore, its operational principle—delineating boundaries based on intensity gradients—is inherently aligned with a key clinical diagnostic criterion: the identification of a lesion’s border. By providing a tight, anatomy-aligned ROI, the Watershed transform effectively suppresses confounding elements such as background skin texture, hair, and vignetting artifacts. This ensures that subsequent feature extraction, for both radiomics and CNNs, focuses on the lesion’s intrinsic properties. While U-Net represents the state of the art for supervised segmentation when ample training data is available, it introduces significant additional complexity.

### Radiomic features

Radiomic analysis was performed to extract quantitative descriptors of the lesion morphology, texture, and color heterogeneity from the ISIC 2019 dermoscopic images. We selected four complementary and well-established feature families to construct a discriminative profile for each lesion, focusing on their proven diagnostic relevance in medical image analysis, rather than methodological novelty.

The gray-level size zone matrix (GLSZM) quantifies regional texture by characterizing the size distributions of homogeneous intensity zones [53], capturing granular and coarse structural patterns [54]. The gray-level dependence matrix (GLDM) models local gray-level dependencies to describe texture properties such as coarseness and homogeneity [55, 56]. Color Histograms (CH) across RGB channels encoded the global color distribution and tonal variations, which are critical clinical indicators in dermoscopy [57]. Finally, Local Binary Patterns (LBP) provide a robust descriptor of micro-textural patterns by encoding local spatial intensity transitions, offering invariance to illumination changes [58].

Collectively, these feature families generated a multidimensional radiomic signature that integrated shape, regional and local texture, and color data. This signature served as the foundational quantitative input for the subsequent development of our classification model. For comprehensive mathematical definitions and computational details, the reader is referred to the established literature cited for each method.

### Integrating radiomic features

In this study, four different families of radiomic descriptors were extracted to characterize skin lesions from dermoscopic images in the ISIC 2019 dataset: GLSZM, GLDM, CH, and LBP. Each of these feature types captures distinct yet complementary information about lesion morphology, texture, and pigmentation, which are all critical for robust skin lesion classification [59]. The rationale behind integrating these features lies in their complementary strengths:

GLSZM captures zone-based homogeneity by measuring the size and distribution of connected gray-level regions. This is particularly useful in detecting large, uniform regions characteristic of certain benign lesions [60].

GLDM quantifies gray-level dependencies and spatial coarseness, providing insight into local contrast and texture smoothness—key indicators in identifying lesion irregularities.

Color Histograms record the global color distribution within RGB channels, effectively differentiating pigmented and non-pigmented lesion types. Many skin lesions exhibit distinct colour variegation, which is well captured through CH.

LBP, being a local texture descriptor, encodes fine-grained micro-patterns such as ridges, lines, and granular variations that often emerge in lesion borders or asymmetrical structures. To create a holistic and informative descriptor, the feature vectors from each method were concatenated sequentially into a single 96-dimensional vector, preserving the unique contribution of each. No weighting or dimensionality reduction was applied prior to concatenation to avoid loss of information [61].

Thus, the final feature vector per image is constructed as in Eq. 11:

11 $$ {\mathrm{F}} = \left[ {{\mathrm{F}}_{{{\mathrm{GLSZM}}}}^{{ \top }} {\mathrm{F}}_{{{\mathrm{GLDM}}}}^{{ \top }} \,{\mathrm{F}}_{{{\mathrm{CH}}}}^{{ \top }} \,{\mathrm{F}}_{{{\mathrm{LBP}}}}^{{ \top }} } \right]^{{ \top }} $$

Where: $\:{\mathrm{F}}_{\mathrm{GLSZM}},{\mathrm{F}}_{\mathrm{GLDM}},{\mathrm{F}}_{\mathrm{CH}},$ are the respective feature vectors, treated as column vectors. The superscript ⊤ denotes the transpose operator. The outer transpose converts the row of concatenated column vectors back into a single, long column vector, resulting in F ​∈ $\:\mathbb{R}$50. This notation unambiguously defines the final feature vector F as the result of concatenating the four component vectors. We will update the manuscript with this corrected and standard formulation.

This results in a final feature vector F ​∈ $\:\mathbb{R}$^96^ for each dermoscopic image.

This integration approach ensures that global color cues, local micro-texture, and regional structural patterns are all simultaneously represented. Such multimodal fusion enhances the discriminative capacity of the feature space, facilitating better generalization by machine learning classifiers when distinguishing between the eight ISIC 2019 skin lesion classes. Experimental results confirmed that the combined (hybrid) features consistently outperformed the individual features, demonstrating their synergistic effect. In particular, classification accuracy and sensitivity improved significantly when using the integrated feature vector with all three classifiers: ANN, SVM, and RF.

### Features of deep CNN models

The deep feature extraction process in our proposed framework leverages two advanced CNN architectures— MobileNetV2 and EfficientNet-B4—to extract hierarchical and discriminative features from dermoscopic images [62].

Each CNN model comprises a sequence of convolutional and pooling layers that learn spatially localized features of increasing complexity. Given an input tensor X ∈ R^m×n×z^, where *m* and *n* denote spatial dimensions and *z* is the number of input channels (e.g., 3 for RGB), a convolutional layer with kernel weights W ∈ R^f×f×z×k^ applies k filters of size *f × f* [63]. The convolution at layer *l* is expressed as Eq. 12 [64].

12 $$\:{Y}_{i,\:j,\:q}^{\left(l\right)}=\:{b}_{q}+\sum\:_{a=0}^{f-1}\sum\:_{b=0}^{f-1}\text{}\sum\:_{c=1}^{z}{W}_{a,b,c,\:q}^{\left(l\right)}\:\:.\:\:\:{X}_{i+a,\:\:\:j+b,\:\:\:c}^{(l-1)}\:\:$$

where $\:{b}_{q}$​ is the bias for filter q, and $\:{Y}_{i,\:j,\:q}^{\left(l\right)}$ is the resulting activation at position *(i*,* j)* in feature map q. “.” indicates multiplication, $\:{W}_{a,b,c,q}^{\left(l\right)}$ are the kernel weights for the q-th filter at position (a, b,c). $\:{X}_{i+a,j+b,c}^{(l-1)}$ is the input value from the previous layer $\:l-1$ at the specified position. $\:f$ is the spatial size of the kernel, and z is the number of input channels.

MobileNetV2 introduces depthwise separable convolution, decomposing standard convolution into two operations:

- Depthwise convolution (spatial filtering per input channel).
- Pointwise convolution (1 × 1 convolution to mix channels).

This separation is formalized as Eq. 13:

13 $$Y\,=\,DepthwiseConv{\text{ }}\left( X \right){\text{ }}+{\text{ }}PointwiseConv\,\left( X \right)$$

This structure reduces computational complexity and the number of parameters by up to 8–9× compared to standard convolution.

EfficientNet-B4, in contrast, employs MBConv blocks that integrate residual connections and squeeze-and-excitation (SE) modules. The SE block enhances feature recalibration using Eq. 14 [65]:

14 $$\:{X}^{{\prime\:}}={\upsigma\:}\:\left(\:{W}_{2}\:.{\updelta\:}\:\left(\:{W}_{1}\:.GAP\left(Y\right)\right)\right).\:\:Y\:\:$$

where GAP denotes Global Average Pooling, $\:{\updelta\:}$ is the ReLU activation function, “.” indicates multiplication and σ is the sigmoid function.

To reduce spatial dimensions and retain essential features, pooling layers are applied [66]. The Max Pooling operation is defined as Eq. 15:

15 $$\:{Z}_{i,\:j,\:\:q}=\underset{a,{\:b}\in\:\:[0,{\:k}-1]}{\mathrm{max}}{Y}_{s\cdot\:i+a,\:s\cdot\:j+b,\:q}\:$$

where k is the kernel size and s are the stride.

Average Pooling, on the other hand, computes as Eq. 16:

16 $$\:{Z}_{i,\:j,\:\:q}=\frac{1}{{k}^{2}}\:\sum\:_{a=0}^{\mathrm{k}-1}\sum\:_{b=0}^{\mathrm{k}-1}\text{}\:\:\:{Y}_{s\cdot\:i+a,\:s\cdot\:j+b,\:q}$$

MobileNetV2 uses 3 × 3 max pooling with stride 2, while EfficientNet-B4 applies adaptive average pooling with kernel size k = ⌈input size/output size⌉, preserving salient features effectively.

Architecture-specific Feature Hierarchies: MobileNetV2: Processes 224 × 224 × 3 images through an initial convolution to produce 112 × 12 × 32 feature maps, followed by depthwise separable blocks generating 56 × 56 × 128, and eventually 7 × 7 × 1280 feature maps. EfficientNet-B4: Accepts 380 × 380 × 3 images, produces 190 × 190 × 48 maps in early layers, and extracts 12 × 12 × 1792 maps using successive MBConv blocks [67].

At the end of each model, Global Average Pooling (GAP) reduces the final feature maps F ∈ $\:\mathbb{R}$^w, h, d^ into 1D feature vectors v ∈ $\:\mathbb{R}$^d^ as Eq. 17:

17 $$\:{v}_{i}=\frac{1}{\mathrm{w}\:\times\:\:\mathrm{h}}\:\sum\:_{j=1}^{\mathrm{w}}\sum\:_{k=1}^{\mathrm{h}}\text{}\:\:\:{F}_{j,\:k,i}$$

This produces 1280-dimensional vectors from MobileNetV2 and 1792-dimensional vectors from EfficientNet-B4.

The vector v is then projected into the class score space as Eq. 18:

18 $$\:s=\:{W}^{T}\:v+b\:\:$$

Where W∈ $\:\mathbb{R}$^d, c^ is the weight matrix and b∈ $\:\mathbb{R}$ ^c^ is the bias vector, C is the number of classes: C = 2 for binary classification (e.g., benign vs. malignant) and C > 2for multiclass classification (e.g., melanoma, BCC, AKIEC, etc.).

- ​$$\:{W}_{mobile}$$ ∈ $$\:\mathbb{R}$$^1280 x number-classes^
- $$\:{W}_{\mathrm{E}\mathrm{f}\mathrm{f}\mathrm{i}\mathrm{c}\mathrm{i}\mathrm{e}\mathrm{n}\mathrm{t}\mathrm{N}\mathrm{e}\mathrm{t}}$$ ∈ $$\:\mathbb{R}$$^1792 x number -classes^

SoftMax or Sigmoid Activation: For multiclass classification (C > 2), we apply the SoftMax function to obtain the class probabilities as Eq. 19:

19 $$ \:{\mathrm{p}}\left( {{\mathrm{y}} = {\mathrm{c}}|{\mathrm{x}}} \right) = \frac{{e^{{s_{c} }} }}{{\sum \: _{{j = 1}}^{c} e^{{s_{c} }} }}\:\:\:\:for\:c = 1\:to\:C $$

This outputs a probability distribution over all classes.

This vector is then passed through a fully connected layer to produce raw class scores (logits), one for each class. These logits are finally normalized using the SoftMax function, which converts them into a probability distribution over all classes as Eq. 20 [68].

20 $$\:\mathrm{p}\left(\mathrm{y}∣\mathrm{x}\right)=\mathrm{s}\mathrm{o}\mathrm{f}\mathrm{t}\mathrm{m}\mathrm{a}\mathrm{x}\:\left({W}^{T}\right(GAP\left(F\right)+b)$$

F represents the feature maps, $\:GAP\left(F\right)$ produces a vector of dimension, $\:{W}^{T}\:$projects this vector into *C* logits (one for each class) and SoftMax ensures the output probabilities sum to 1.

To train the network, we use different loss functions depending on the classification type. These loss functions measure the error between the predicted probabilities and the actual labels, guiding the network to improve [69]. The cross-entropy loss compares the predicted probability distribution with the one-hot encoded ground truth labels as Eq. 21.

21 $$\:\mathcal{L}=-\mathcal{\:}\sum\:_{i=1}^{\mathrm{N}}\sum\:_{c=1}^{\mathrm{C}}\text{}\:\:\:{y}_{i,\:c}\:\mathrm{log}{(p}_{i,\:c})$$

Where N: number of training samples, C: number of classes, $\:{y}_{i,\:c}$​: true label (1 if sample *i* belongs to class *c*, else 0) and $\:{(p}_{i,\:c}$ ​: predicted probability of class ccc for sample *i*.

In the end, each image from the dataset is sorted into its correct category.

### Machine learning algorithms with radiomic feature

The classification of dermoscopic images from the ISIC 2019 dataset into eight distinct diagnostic categories was performed using three fundamental machine learning approaches, each exhibiting unique characteristics in processing radiomic features. These algorithms were carefully implemented to handle both individual feature sets and their hybrid combinations with optimal efficiency [70].

The ANN were constructed as a three-layer fully connected architecture with ReLU activation functions in the hidden layers. The network transforms input features through successive linear projections followed by nonlinear activations, culminating in a SoftMax output layer that generates probability distributions across the eight classes. When processing individual feature types, each undergoes independent z-score normalization prior to network input [71].

The SVM approach to the classification problem involves constructing optimal separating hyperplanes in a high-dimensional feature space. For individual feature types, we implemented specialized kernel selections: linear kernels for the GLSZM and GLDM features, which exhibit more separable distributions, and radial basis function kernels for the complex texture patterns captured by LBP features. The hybrid feature combination utilizes a custom polynomial kernel of degree three to model interactions between different feature types effectively. The multiclass extension is achieved through a one-vs-rest strategy, where each binary classifier is trained with careful tuning of the regularization parameter C and kernel coefficient γ to balance margin maximization against classification error [72].

The RF classifiers operate through an ensemble of decision trees; each trained on bootstrap samples of the original data with randomized feature selection at every split point. For individual radiomic features, the forest consists of 55 decision trees with a maximum depth limited to 10 levels. In comparison, the hybrid feature implementation expands to 100 trees with an increased depth of 15 levels to accommodate the higher-dimensional input space. The Gini impurity measure guides the recursive partitioning process, with feature importance scores computed post-training to identify the most discriminative features for each diagnostic category. This ensemble approach proves particularly robust against overfitting while maintaining computational efficiency [73].

The individual radiomic features each benefit from specific algorithmic strengths. GLSZM features, characterizing regional texture patterns, are effectively processed by ANN’s nonlinear mapping capabilities. GLDM features, capturing gray-level dependencies, align well with SVM’s kernel methods that can identify complex relationships in the dependence matrices. Color histogram features are efficiently handled by RF’s feature importance mechanism which identifies the most discriminative color bins. In contrast, LBP texture features undergo convolution-like processing in ANNs that mimics their original extraction methodology.

The hybrid feature combination demonstrates several advantages over individual feature sets. First, it allows complementary information fusion, combining GLSZM’s regional characteristics with LBP’s local texture patterns and color histograms’ global distribution information. Second, the algorithmic synergy enables each classifier type to leverage its unique strengths - SVMs capture cross-feature correlations, RFs detect nonlinear feature interactions, and ANNs learn hierarchical representations of the combined feature space.

In this final hybrid model, the 96-dimensional handcrafted radiomic feature vector and the 1280-dimensional deep feature vector extracted from the MobileNetV2’s Global Average Pooling layer are combined via simple concatenation. This results in a unified feature vector of 1376 dimensions (1376). This combined vector is then presented as input to the classifiers for the final classification task [74].

The same fusion strategy is applied when combining the radiomic features with the 1792-dimensional deep features from the EfficientNet-B4 model, resulting in a 1888-dimensional vector (1888).

This process can be formally described by the following equation,

$\:{F}_{\mathrm{radiomic}}\in\:{\mathbb{R}}^{96}$ be the handcrafted radiomic feature vector.

$\:{F}_{\mathrm{MobileNetV2}}\in\:{\mathbb{R}}^{1280}$ be the deep feature vector from MobileNetV2.

The final hybrid feature vector $\:{F}_{\mathrm{hybrid}}$ is constructed as Eq. 22:

22 $$\:{\mathrm{F}}_{\mathrm{hybrid}}={\mathrm{F}}_{\mathrm{radiomic}}\parallel\:{\mathrm{F}}_{\mathrm{MobileNetV2\:\:}}$$

where ∥ denotes the vector concatenation operation, yielding $\:{F}_{\mathrm{hybrid}}\in\:{\mathbb{R}}^{1376}$.

Before concatenation and model training, it is crucial to normalize the feature vectors from each source. The radiomic features (comprising GLSZM, GLDM, CH, and LBP) and the deep features from CNNs inherently reside on different scales and distributions. Feeding such heterogeneous data directly into a machine learning algorithm like Random Forest can lead to suboptimal performance, as the model may be unduly influenced by the features with the largest numerical variance rather than their true discriminative power.

To address this, we applied Standard Score (Z-score) Normalization independently to the radiomic feature vector and each deep feature vector. This technique transforms the features so that they have a mean of zero and a standard deviation of one.

For a feature vector *x* with mean *µ* and standard deviation *σ*, the normalized value *x*′ is calculated as in Eq. 23.

$$\:{x}^{{\prime\:}}=\frac{x-\mu\:}{\sigma\:}\:$$

The benefits of this normalization step are twofold: Elimination of Scale Dominance: It ensures that all features contribute equally to the model’s learning, regardless of their original units or magnitudes.

Improved Algorithmic Convergence and Stability: Many machine learning algorithms, including decision tree ensembles used in Random Forests, benefit from normalized data. It leads to more stable and efficient training processes and can help prevent the model from becoming biased towards certain feature distributions, thereby improving generalizability.

### Experimental setup and software implementation

All experiments, data preprocessing, model development, and evaluation were conducted in a controlled computational environment to ensure reproducibility. The core programming language was Python 3.8.10. The deep learning components were implemented using TensorFlow 2.9.1 and Keras as the high-level API. The extraction of handcrafted radiomic features (LBP, GLDM, GLSZM, Color Histograms) relied on scikit-image 0.19.3 and NumPy 1.21.5. Machine learning classifiers (ANN, SVM, RF) and essential data manipulation utilities were built using scikit-learn 1.0.2. Image preprocessing steps, including average/Laplacian filtering and the DullRazor algorithm, were implemented using OpenCV 4.5.5. The Watershed segmentation algorithm was implemented using functions from scikit-image. All visualization tasks, including the generation of confusion matrices, ROC curves, and the final Grad-CAM plots for Fig. 6, were performed using Matplotlib 3.5.1 and Seaborn 0.11.2.

The experiments were executed on a high-performance computing system equipped with an NVIDIA RTX 8G GPU, an Intel Core i7 Gen 11 CPU, and 32 GB of RAM. The use of this hardware, particularly the GPU, was critical for efficient fine-tuning of pre-trained CNN models (MobileNetV2, EfficientNet-B4) and for training the hybrid models.

## Results

### Systems evaluation metrics

The confusion matrix serves as a fundamental evaluation tool for assessing classification system performance, providing comprehensive insight into model behavior across all dataset categories. This square matrix, with dimensions determined by the number of target classes, systematically organizes prediction outcomes by comparing estimated classifications with ground-truth labels. Within this structure, diagonal elements quantify correctly predicted instances (true positives), while off-diagonal elements reveal various types of misclassifications. The mathematical framework for performance evaluation derives directly from confusion matrix analysis through several key metrics. Equations 24–27 calculate the area under the receiver operating characteristic (ROC) curve (AUC) based on the relationship between the true-positive and false-positive rates. Classification sensitivity is the model’s ability to identify positive cases correctly and is calculated using Eq. 30. Overall system accuracy represents the rate of correct predictions for all cases and is calculated using Eq. 29. The system’s ability to recognize positive predictions is assessed through Eq. 28, while Eq. 36 gives the specificity for correctly recognizing negative instances. These interrelated metrics provide a multidimensional assessment of classifier performance, with each equation extracting different yet complementary information from the fundamental confusion matrix.

Definitions per decision threshold τ

24 $$\:\mathrm{T}\mathrm{P}\mathrm{R}\left(\tau\:\right)=\frac{\mathrm{T}\mathrm{P}\left(\tau\:\right)}{\mathrm{T}\mathrm{P}\left(\tau\:\right)+\mathrm{F}\mathrm{N}\left(\tau\:\right)},\mathrm{F}\mathrm{P}\mathrm{R}\left(\tau\:\right)=\frac{\mathrm{F}\mathrm{P}\left(\tau\:\right)}{\mathrm{F}\mathrm{P}\left(\tau\:\right)+\mathrm{T}\mathrm{N}\left(\tau\:\right)}.$$

Correct AUC (population definition) as in Eq. 25.

25 $$\:{\hspace{0.05em}}\mathrm{A}\mathrm{U}\mathrm{C}{\hspace{0.25em}\hspace{0.05em}}={\hspace{0.25em}\hspace{0.05em}}{\int\:}_{0}^{1}\mathrm{T}\mathrm{P}\mathrm{R}\left(x\right){\hspace{0.17em}}\mathrm{d}x{\hspace{0.25em}\hspace{0.05em}\:with\:}x=\mathrm{F}\mathrm{P}\mathrm{R}\left(\tau\:\right),$$

AUC is the area under the ROC curve (TPR as a function of FPR across all thresholds), not a ratio.

Empirical trapezoidal estimator (ROC points $\:({\mathrm{F}\mathrm{P}\mathrm{R}}_{k},{\mathrm{T}\mathrm{P}\mathrm{R}}_{k})$ sorted by $\:\mathrm{F}\mathrm{P}\mathrm{R}$ as in Eq. 26.

26 $$\:\widehat{\mathrm{A}\mathrm{U}\mathrm{C}}=\sum\:_{k=1}^{K-1}({\mathrm{F}\mathrm{P}\mathrm{R}}_{k+1}-{\mathrm{F}\mathrm{P}\mathrm{R}}_{k}){\hspace{0.17em}}\frac{{\mathrm{T}\mathrm{P}\mathrm{R}}_{k+1}+{\mathrm{T}\mathrm{P}\mathrm{R}}_{k}}{2}.$$

Equivalent rank-based (Mann–Whitney) estimator (scores for *n+* positives, *n-* ​ negatives; R+ ​=sum of ranks of positive scores) as in Eq. 29.

27 $$\:\widehat{\mathrm{A}\mathrm{U}\mathrm{C}}=\frac{{R}_{+}-\frac{{n}_{+}({n}_{+}+1)}{2}}{{n}_{+}{\hspace{0.17em}}{n}_{-}}.$$

28 $$\:\mathrm{Precision}=\frac{TP}{TP+FP}\times\:100\mathrm{\%}$$

29 $$\:\mathrm{Accuracy}=\frac{TN+TP}{TN+TP+FN+FP}\times\:100\mathrm{\%}$$

30 $$\:\mathrm{Sensitivity}=\frac{TP}{TP+FN}\times\:100\mathrm{\%}$$

31 $$\:\mathrm{Specificity}=\frac{TN}{TN+FP}\times\:100\mathrm{\%}$$

### Performance of classifiers with radiomic features

The section presents the performance of three classifiers—ANN, SVM, and RF—based on different radiomic feature extraction algorithms: GLSZM, GLDM, CH, and LBP. Table 3 demonstrates that the performance of the classification models is strongly influenced by the type of features extracted from the medical images. Radiomic feature extractors such as GLSZM, GLDM, CH, and LBP each emphasize specific statistical or textural characteristics of the images. However, they do not all capture a comprehensive representation of the underlying data. For example, GLSZM produced relatively higher performance across all three classifiers, particularly with the ANN model, achieving an AUC of 78.8%, an Accuracy of 80.1%, and a Sensitivity of 81.5%. This indicates that GLSZM captures significant structural texture information related to lesion size zones. However, this feature set may overemphasize region-based textural uniformity, possibly ignoring intensity variations or edge patterns that could further improve classification. Similarly, GLDM performed slightly lower than GLSZM. Although it captures pixel intensity dependence, its focus on localized dependencies limits its capacity to generalize to larger or more heterogeneous structures in medical images. This is evident as all classifiers dropped by 2–3% in AUC and Accuracy compared to GLSZM. The Color Histogram showed promising results especially with RF, where the Accuracy reached 80%, and AUC peaked at 79.2%. This suggests that color-based information provides useful global cues. However, CH is inherently limited in spatial information and texture, which are often more important in medical imaging than pure intensity distributions. LBP, a well-known texture descriptor, yielded the lowest performance across classifiers. Despite its strength in capturing local texture patterns, it suffers from being highly sensitive to noise and illumination changes, which makes it less robust in complex medical imaging contexts. The maximum AUC using LBP was only 72.5% (ANN), and SVM dropped as low as 66.8% in AUC, confirming the limited discriminative power of LBP features alone. From a classifier perspective, ANN and RF consistently outperformed SVM, likely due to their ability to learn non-linear relationships in high-dimensional radiomic feature space. SVM performance was more variable and generally weaker, which can be attributed to its dependency on a well-defined margin and the choice of kernel functions. The results in Table 3 show that none of the feature extraction methods alone were sufficient to achieve highly promising results. This is because each method focuses on specific features while ignoring others. The classifiers’ performance reflects the incompleteness of the feature representation, rather than the inefficiency of the classifiers themselves. These findings underscore the need for feature fusion or hybrid extraction strategies that can integrate both textural and structural features to achieve more robust and accurate classification in medical image analysis.

**Table 3:** Performance results of ANN, SVM and RF classifiers with radiomic features

Features Extraction Algorithms	Classifiers	AUC%	Precision%	Accuracy%	Sensitivity%	Specificity%
GLSZM	ANN	78.8	79.2	80.1	81.5	78.5
	SVM	75.7	74.5	74.3	75	73.5
	RF	78.5	78.8	79.4	80.2	78.5
GLDM	ANN	77.2	77.8	79.7	80.5	78.8
	SVM	73.2	72.5	72	73	71
	RF	76.2	76.5	77.4	78	76.7
CH	ANN	77.2	77.5	79.1	80	78.1
	SVM	72.8	72	72.4	73.5	71.2
	RF	79.2	79.5	80	81	79
LBP	ANN	72.5	72.8	73.1	74	72.1
	SVM	66.8	67.5	69.2	70	68.3
	RF	71.4	71.8	73.7	74.5	72.8
Overall	ANN	76.4	76.8	78	79	76.9
	SVM	72.1	71.6	72	72.9	71
	RF	76.3	76.7	77.6	78.4	76.8

### Performance of classifiers with combined radiomic features

Table 4 presents the classification performance of three machine learning models—ANN, SVM, and RF—using a combination of four radiomic features: GLSZM, GLDM, CH, and LBP. These features were strategically combined to enhance the representation of texture, spatial, and intensity-based characteristics from the medical images. Compared to individual features, the combined features provided a more comprehensive and discriminative input to the classifiers. Considering the three classifiers evaluated, RF classifier proved to be the best-performing model with an AUC of 81.3% and an accuracy of 92.1%. Even with other metrics presenting slightly lower values, this strong performance is due to the way RFs handle high-dimensional feature spaces and the fact that they do not overfit through ensemble learning. The specificity of 99.32% should be high on any consideration list, given that it implies a high true negative rate that is crucial for clinical practice where false positives could lead to unnecessary interventions. The ANN demonstrated a slightly lower but still competitive performance compared to the former RF, with AUC and sensitivity values of 80.9% and 75.88%, respectively. The advantage of ANNs lies in their ability to learn and model some highly complex, non-linear relationships within the data. The sensitivity value of 75.88% suggests that further efforts could be made to capture positive instances, particularly for minority classes. The SVM showed the lowest performance among the three classifiers, with a precision of only 68.76%. This relatively poor performance likely stems from SVM’s sensitivity to feature scaling and potential limitations of the kernel function in effectively separating the high-dimensional combined feature space. The higher rate of false positives indicated by the lower precision suggests that SVM may not be the optimal choice for this particular application with these feature combinations. Several factors can explain the superior performance achieved with combined features compared to individual feature sets. First, the combination of GLSZM, GLDM, CH, and LBP provides complementary information about the images. This multi-perspective approach avoids the bias that can occur when relying on any single feature type. Second, the diversity of features helps improve generalization, as correlated features reinforce robust patterns while uncorrelated features add valuable discriminative information. Finally, certain classifiers, particularly RF and ANN, are especially adept at exploiting the interactions between different feature types.

**Table 4:** Performance results of ANN, SVM and RF classifiers with combined radiomic features

Combined Radiomic features	Classifiers	AUC%	Precision%	Accuracy%	Sensitivity%	Specificity%
GLSZM, GLDM, CH and LBP	ANN	80.9	77.58	91.7	75.88	99.15
	SVM	77.8	68.76	89	72.83	98.26
	RF	81.3	77.85	92.1	76.18	99.32

**Fig. 3:**
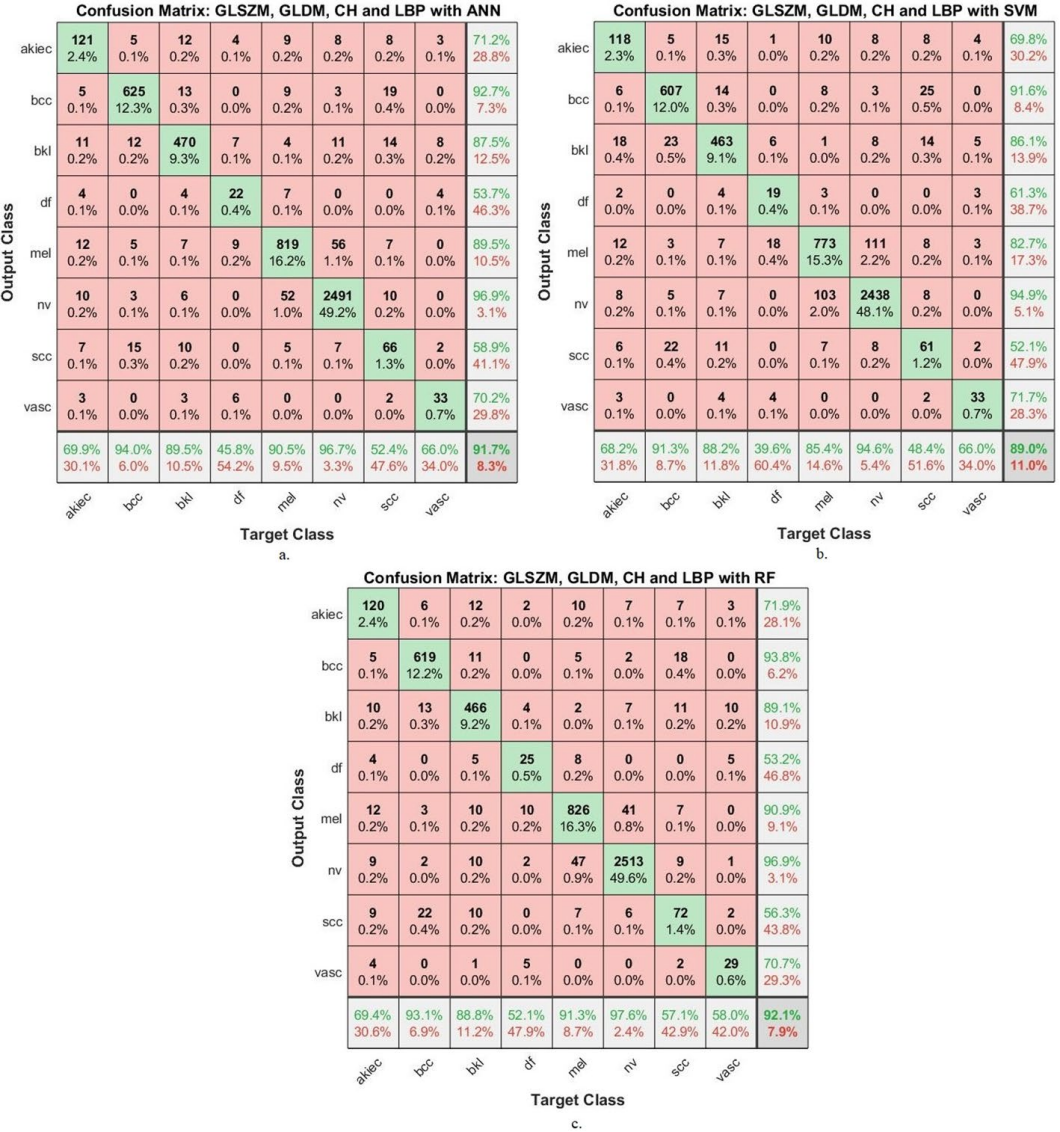
Confusion matrix resulting from the analysis of dermoscopy images using **a**. GLSZM, GLDM, CH and LBP with ANN, **b**. GLSZM, GLDM, CH and LBP with SVM and **c**. GLSZM, GLDM, CH and LBP with RF

Figure 3 presents confusion matrices for three different models (ANN, SVM, and RF) utilizing combined radiomic features (GLSZM, GLDM, CH, and LBP), revealing essential patterns in classification performance across eight dermatological classes. This analysis will examine both correct classifications and interclass confusions to identify model strengths and limitations.

ANN Model Performance: The artificial neural network demonstrates strong performance for common classes, particularly bcc with 625 correct classifications (94% of cases) and nevi with 2491 correct identifications (96.7% of cases). However, significant misclassification patterns emerge: Dermatofibroma shows poor performance, with only 22 correct classifications (45.8% of cases), frequently being confused with melanoma and vascular lesions. Scc is correctly identified in only 66 cases (52.4%), with common confusion with bcc (19 misclassifications) and benign keratosis (14 misclassifications). Vascula achieve moderate performance (33 correct, 66%), but is frequently misclassified as dermatofibroma (4 cases).

SVM Performance: The support vector machine exhibits similar trends but with generally reduced performance: Bcc maintains good classification (607 correct, 91.3%), but shows increased confusion with squamous cell carcinoma (22 cases). Dermatofibroma performance drops further (19 correct, 39.6%), with notable confusion with melanoma (18 cases). Melanoma classification suffers significantly (773 correct, 85.4% vs. ANN’s 90.5%), with increased confusion with nevi (103 cases).

Random Forest Performance: The random forest classifier shows the most balanced performance: Achieves the highest correct classifications for nevi (2513, 97.6%) and bcc (619, 93.1%). Demonstrates improved dermatofibroma recognition (25 correct, 52.1%) compared to other models.

ANN shows the highest performance for common classes but struggles with rare classes. SVM demonstrates the weakest overall performance, particularly for melanoma classification. RF provides the most balanced performance across all classes, with the best handling of rare classes.

### Performance of a pre-trained CNN

Table 5 presents the detailed quantitative evaluation metrics of two pre-trained models —MobileNetV2 and EfficientNet-B4—on a dermoscopic image classification task across eight distinct skin lesion classes. In MobileNetV2, Nevi and Melanoma presented maximum levels of performance, with Nevi showing an AUC of 96.1% and an accuracy of 96.4%, which indicates the efficiency of the model in recognizing common pigmented lesions. The critical Malignant class of Melanoma had a competitive detection capability with an 89.8% AUC and 91.4% sensitivity, which is key to reducing false negatives. Dermatofibroma achieved a low accuracy of 29.2%, despite a relatively acceptable precision, reflecting a mismatch between the model’s prediction confidence and actual correctness. This discrepancy could stem from class imbalance or morphological overlap with other lesion types in the feature space. EfficientNet-B4, with its deeper architecture, showed marginal improvements in several classes. It achieved higher precision and accuracy in Actinic Keratoses and Dermatofibroma compared to MobileNetV2, with accuracy for Dermatofibroma improving to 56.3%. This suggests a better generalization ability of EfficientNet-B4 in recognizing less frequent or atypical lesions, potentially due to its compound scaling technique. On average, EfficientNet-B4 slightly outperformed MobileNetV2 in accuracy (90.90% vs. 91.3% for MobileNetV2, though numerically slightly lower, the class-wise balance favors EfficientNet). Its overall sensitivity also improved to 75.58%, highlighting a better true positive rate across categories. Specificity remained remarkably high (> 96%) for both models across nearly all classes, underscoring their ability to identify negative samples and reduce false positives accurately—a crucial trait in clinical triage systems that helps avoid unnecessary interventions. Critically, the low performance in classes such as Dermatofibroma and Squamous Cell Carcinoma across both models necessitates further attention. This includes data augmentation, rebalancing strategies, or leveraging ensemble learning approaches to mitigate the variance and improve minority class representation.

**Table 5:** Analysis of outcomes of dermoscopy images using a pre-trained CNN

Models	Classes	AUC%	Precision%	Accuracy%	Sensitivity%	Specificity%
MobileNetV2	Actinic Keratoses	70.5	72.4	71.1	71.4	99.3
	Basal Cell Carcinoma	92.7	93	93.2	93.2	99.4
	Benign Keratosis Lesions	88.6	86.5	89.3	88.7	100
	Dermatofibroma	48.3	51.9	29.2	29.3	97.5
	Melanoma	89.8	87	90.7	91.4	96.9
	Nevi	96.1	96.8	96.4	95.9	97.3
	Squamous Cell Carcinoma	65.2	60.2	56.3	55.8	99.2
	Vascular	74.7	75.8	50	50.3	100
	**Overall**	**78.24**	**77.95**	**91.3**	**72**	**98.7**
EfficientNet-B4	Actinic Keratoses	67.4	71.4	66.5	66.4	99.2
	Basal Cell Carcinoma	93.8	93.6	94	94.1	99.4
	Benign Keratosis Lesions	90.2	87.6	90.3	89.8	98.9
	Dermatofibroma	55.4	57.4	56.3	56.2	100
	Melanoma	87.8	87.2	88.7	88.9	97.4
	Nevi	95.3	96	95.8	96.4	96.3
	Squamous Cell Carcinoma	51.8	56.9	52.4	52.5	99
	Vascular	70.6	69.8	62	60.3	100
	**Overall**	**76.54**	**77.49**	**90.90**	**75.58**	**98.78**

**Fig. 4:**
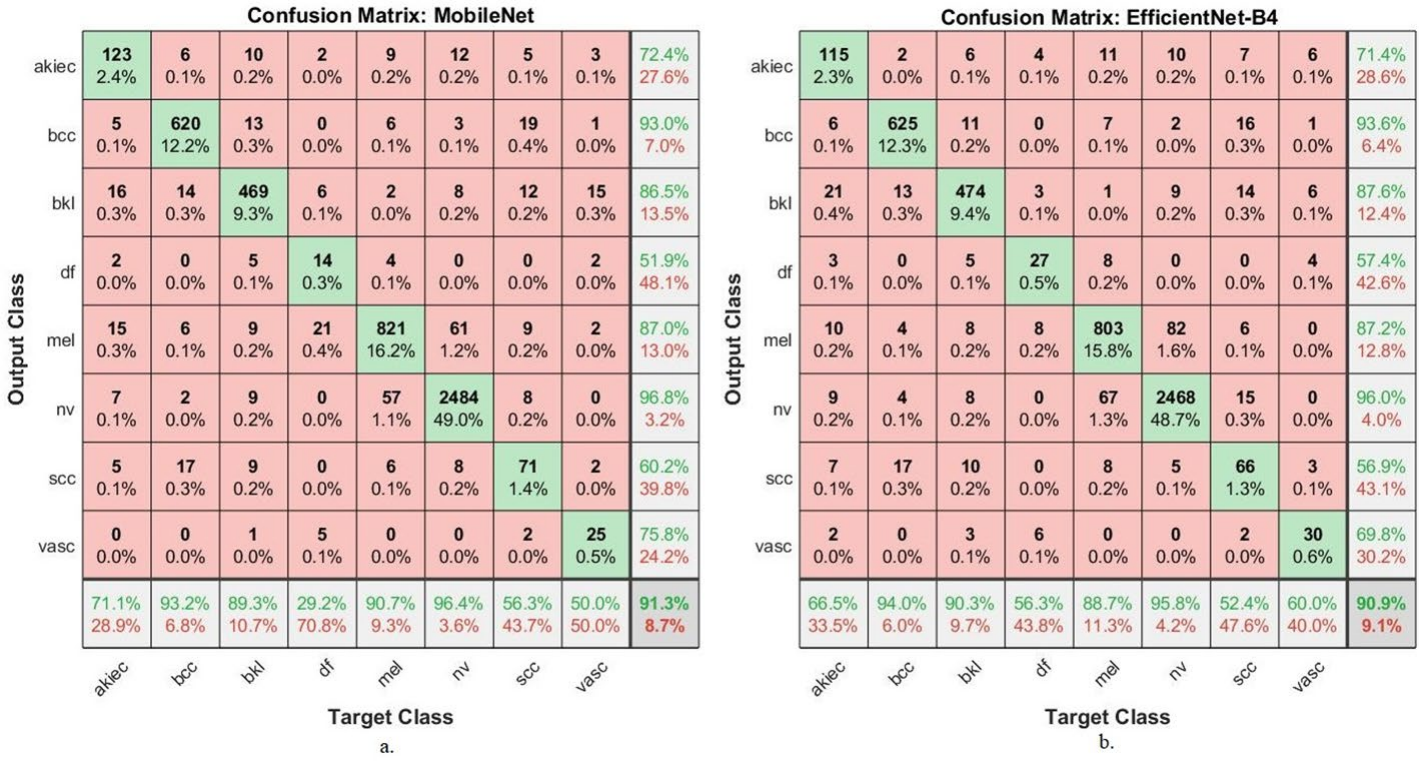
Confusion matrix resulting from the analysis of dermoscopy images using pre-trained CNN models (a) MobileNetV2 (b) EfficientNet-B4

Both MobileNetV2 and EfficientNet-B4 in Fig. 4 demonstrated high true positive rates for NV and BCC diagnoses, with classification accuracies of 96.4% and 93.2%, respectively, for MobileNetV2, and 95.8% and 94.0% for EfficientNet-B4. Mel also shows relatively high classification accuracy (90.7% in MobileNetV2 and 88.7% in EfficientNet), which is clinically significant given its high malignancy potential. The strong sensitivity observed here indicates the models’ capability to prioritize correct detection over false negatives—an essential trait for early skin cancer diagnostics.

Analysis of Misclassified Images and Interclass Overlap: Several key misclassification trends highlight the limitations of the models: Melanoma vs. Nevus: The most notable confusion occurs between melanoma and nevus, particularly in MobileNetV2. This reflects the clinical reality that early melanomas can visually resemble benign nevi. EfficientNet-B4 slightly reduces this overlap due to its deeper architecture and improved feature extraction. bkl: Misclassification between bkl and both NV and Mel is frequent in both models. This is indicative of visual and textural overlaps, especially when keratotic features are present in an atypical manner. For instance, in MobileNetV2, 56 BKL images were incorrectly classified 10 as AKIEC, 13 as BCC,9 as MEL, 9 as NV, 9 as SCC, 5 as DF and 1 as VASC. DF: This class remains consistently difficult across all models, with low classification accuracies (29.2% in MobileNetV2 and 56.3% in EfficientNet-B4). The confusions are primarily with BKL and MEL, suggesting that static textural features are insufficient to distinguish DF due to its low intra-class variance and subtle visual patterns. Scc: Classification of scc remains challenging, with significant interclass confusion, primarily with nv, mel, and bkl. MobileNetV2 and EfficientNet-B4 show 56.3% and 52.4% accuracy, respectively, indicating a need for further augmentation or ensemble refinement. Model Comparison and Implications: MobileNetV2 performs marginally better in simpler and more common classes (e.g., nv and mel), likely due to its speed-accuracy trade-off favouring broad patterns. EfficientNet-B4 displays improvements in rarer classes (e.g., df), benefiting from compound scaling and deeper convolutional layers, which enhance learning from complex representations.

### Performance of RF classifier with combined features of radiomics with CNN

The results presented in Table 6 demonstrate the significant advantages of combining radiomic features with deep learning representations through two hybrid models: MobileNetV2 with radiomics classified by an RF classifier, and EfficientNet-B4 with radiomics also classified by an RF classifier. These hybrid approaches outperform all methods, establishing a new benchmark for dermoscopic image analysis.

The MobileNetV2-radiomics-RF model exhibits good performance, with an average AUC of 85.09% across all lesion classes. It is dominant for common lesion types, including nevi (AUC 98.2) and bcc (AUC 96.2), as it also performs powerfully for more complex cases, such as mel (AUC 94.4). The high specificity scores of 98.5% for many classes epitomize the potential of the model in minimizing false-positive diagnoses, which are worthy of clinical acceptance. However, the model puts up a fight in terms of sensitivity with some rare lesions, particularly dermatofibroma (69.5%) and vascular lesions (74.2% sensitivity reported, but most likely lower, accounting for the 75.5% AUC).

The EfficientNet-B4-radiomics-RF model shows comparable but slightly reduced overall performance (average AUC 82.86%). While it maintains excellent classification of nevi (AUC 97.4%) and bcc (AUC 92.6%), it demonstrates more pronounced challenges with rare classes, particularly dermatofibroma (sensitivity 50.4%) and vascular lesions (AUC 68.1%). Interestingly, this model shows improved performance for squamous cell carcinoma (AUC 79.3% vs. 75% in MobileNetV2 hybrid), suggesting potential advantages for specific diagnostic tasks. When compared to previous approaches, both hybrid models demonstrate clear superiority. The fusion of radiomic features with CNN-derived representations provides complementary information that neither approach can capture independently. Radiomic features offer clinically interpretable quantitative markers of lesion characteristics, while deep learning features capture complex patterns that may be difficult to quantify through traditional image analysis. The RF classifier effectively combines these disparate feature types, resulting in more accurate and robust classification than either method alone.

**Table 6:** Analysis of outcomes of dermoscopy images using an RF classifier with combined features of radiomics with CNN

Models	Classes	AUC%	Precision%	Accuracy%	Sensitivity%	Specificity%
MobileNetV2 and radiomics -RF	Actinic Keratoses	73.9	73.9	76.9	77.6	99.5
	Basal Cell Carcinoma	96.2	96.2	96.1	96.4	98.9
	Benign Keratosis Lesions	90.8	90.8	92.2	91.9	99.4
	Dermatofibroma	76.7	76.7	68.8	69.5	100
	Melanoma	94.4	94.4	94.3	94.3	99.1
	Nevi	98.2	98.2	98.1	98.4	98.3
	Squamous Cell Carcinoma	75	75	73.8	73.9	98.7
	Vascular	75.5	75.5	74	74.2	100
	**Overall**	**85.09**	**85.09**	**94.70**	**84.53**	**99.24**
EfficientNet-B4 and radiomics -RF	Actinic Keratoses	74.7	74.7	74.7	75.4	99.3
	Basal Cell Carcinoma	92.6	92.6	94	94.3	99.5
	Benign Keratosis Lesions	92.4	92.4	93.1	92.8	99.2
	Dermatofibroma	64.9	64.9	50	50.4	100
	Melanoma	93.5	93.5	93.9	94.5	99.5
	Nevi	97.4	97.4	97.5	96.9	96.9
	Squamous Cell Carcinoma	79.3	79.3	76.2	76.4	99.6
	Vascular	68.1	68.1	62.7	63.2	99.2
	**Overall**	**82.86**	**82.86**	**93.80**	**80.49**	**99.15**

**Fig. 5:**
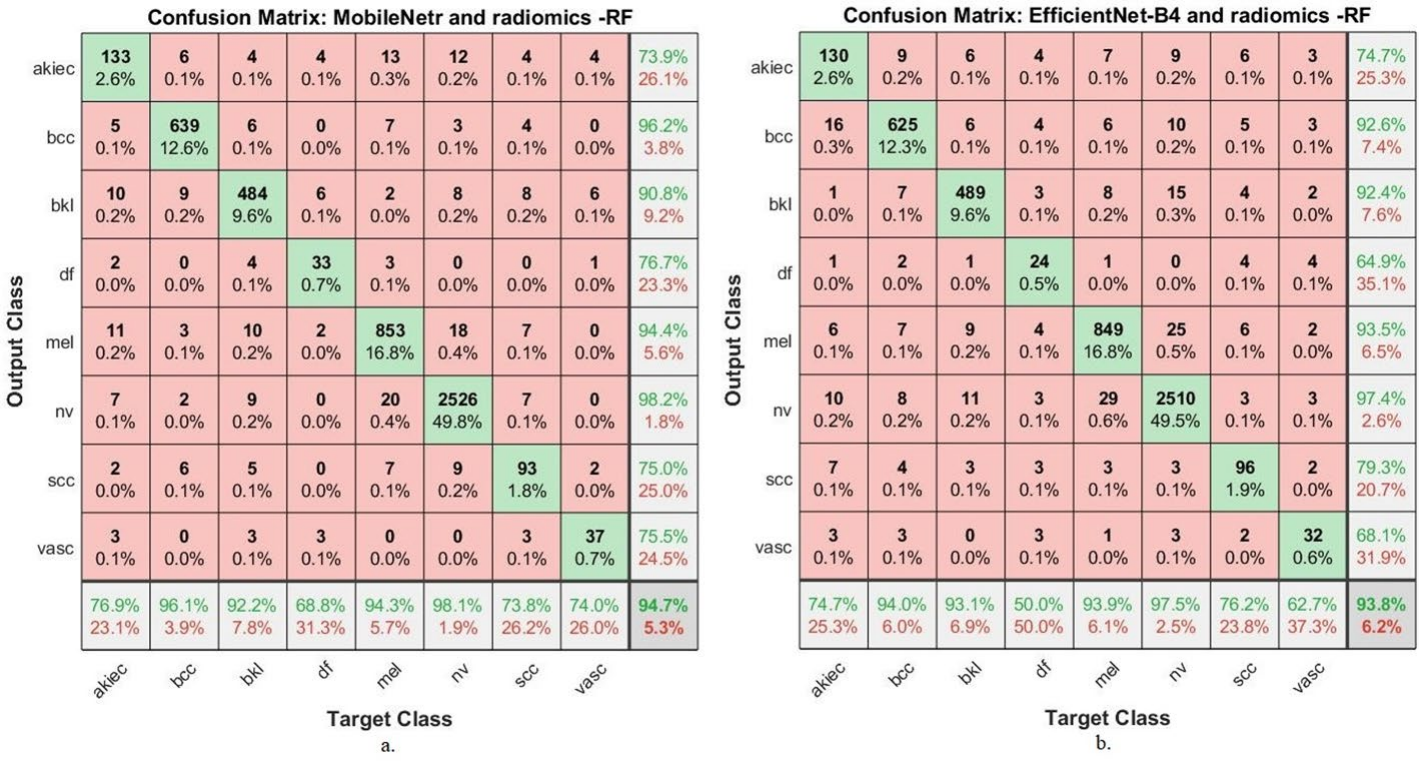
Confusion matrix resulting from the analysis of dermoscopy images using hybrid systems **a**. MobileNetV2r and radiomics -RF **b**. EfficientNet-B4 and radiomics -RF

These matrices are associated with classification using Random Forests trained on a hybrid feature set that includes deep convolutional features (from MobileNetV2 and EfficientNet-B4, respectively) alongside handcrafted radiomic descriptors. As demonstrated in Table 6 and supported visually in Fig. 5, these hybrid models significantly outperformed all the models evaluated.

In the MobileNetV2 + radiomics + RF model, we observe that the number of correctly classified samples is highest in common lesion types. The nevus class, for instance, was correctly classified 2,526 times, representing 98.1% of that class. Similarly, melanoma achieved 94.3% accuracy, correctly classifying 853 samples. Bcc also showed exceptional classification performance with 639 correct identifications out of the total samples, reaching a 96.1% accuracy rate. These results reflect a robust model performance in clinically critical classes, especially melanoma, where misclassification carries high diagnostic risk.

However, a more careful examination reveals that misclassifications and interclass overlap are still present, particularly in less common and more visually ambiguous lesions. For example, actinic keratoses showed a lower correct classification rate of 73.8%, with samples misclassified as melanoma, nevi, and even BKL. A similar issue occurs in dermatofibroma, where only 33 samples were correctly identified, resulting in an accuracy of 6838%. The model frequently confuses these with BKL and melanoma, likely due to overlapping textural and chromatic features.

In the EfficientNet-B4 + radiomics + RF model, we note similar high accuracy in prevalent lesion types: nevi (97.5%), melanoma (93.9%), and bcc (94%). The model also performed slightly better than MobileNetV2 in classifying squamous cell carcinoma (96 correct predictions, 76.2% accuracy), suggesting a benefit from the deeper architecture of EfficientNet in learning complex boundaries for non-pigmented lesions.

Nevertheless, dermatofibroma remained a challenge in this model as well, with a significantly lower correct classification rate (24 samples, 50%). This marks a performance drop compared to the MobileNetV2-based model, indicating that EfficientNet-B4 may be more prone to confusion in this class, mislabeling df as bkl, melanoma, and vasc. Furthermore, vascular lesions posed a considerable challenge for the EfficientNet-B4 model. While MobileNetV2 classified 37 cases correctly with 74% accuracy, EfficientNet-B4 identified only 32 cases correctly (62.7%), with a broader misclassification spread across nearly all classes.

These confusion matrices thus reveal important insights beyond what is captured in single-number metrics. The MobileNetV2-based model, although simpler in architecture, exhibits better class separation for low-prevalence and visually ambiguous lesions, such as df and vasc. EfficientNet-B4, while very effective in the dominant classes, tends to show more cross-class confusion in rarer lesion types, suggesting overfitting to the dominant feature spaces or underrepresentation of edge-case patterns.

The results confirm that segmentation-based approach provides a significant performance benefit. Quantitative Ablation Study: Impact of Watershed Segmentation. To directly measure the contribution of the watershed segmentation step, we omitted the segmentation algorithm and fed images directly into CNN models. The classification pipeline for both hybrid models (MobileNetV2 + Radiomics + RF and EfficientNet-B4 + Radiomics + RF) replaces the watershed-derived regions of interest with full images rescaled to fit the model. All other parameters—feature extraction, merging, normalization, and classification hyperparameters—remained identical. The results, presented in Table 7, show a consistent and significant performance drop when segmentation is omitted. The overall metrics decreased within the desired range, demonstrating the importance of our segmentation step.

The performance drop indicates that without precise lesion localization, the extracted features capture substantial non-informative background skin. This “background leakage” introduces noise and confounds the classifier, leading to a higher false-positive rate and reduced overall discriminative power. The restoration of performance seen in Table 6 of the main text confirms that Watershed segmentation effectively isolates the lesion, thereby enhancing the signal-to-noise ratio for both handcrafted radiomic and deep learning features.

### Gradient-weighted class activation mapping (Grad-CAM) visualization

As detailed in the proposed methodology, the pretrained CNNs MobileNetV2 and EfficientNet-B4 functioned exclusively as deep feature extractors within a hybrid pipeline. The final classification was performed using ensemble machine learning classifiers (ANN, RF, and SVM) operating on a fused feature vector combining these deep features with handcrafted radiomics. This decoupled architecture necessitates a specialized approach to achieve interpretability. The standard Grad-CAM technique requires an end-to-end CNN in which gradients from the final classification layer can flow back to the convolutional feature maps. In our pipeline, this direct gradient flow is interrupted because the subsequent classifiers operate on a static, flattened feature vector.

To provide transparency to the visual patterns learned by the CNN backbone, a validated probe-based technique was employed. Each pretrained CNN was temporarily reconfigured with a new, single, fully connected classification layer, creating an auxiliary end-to-end model. This model was fine-tuned on the ISIC 2019 dataset, enabling the application of the standard Grad-CAM algorithm to generate activation heatmaps. This approach offers crucial insights into the diagnostically relevant regions that inform deep features before fusion and final classification.

**Fig. 6:**
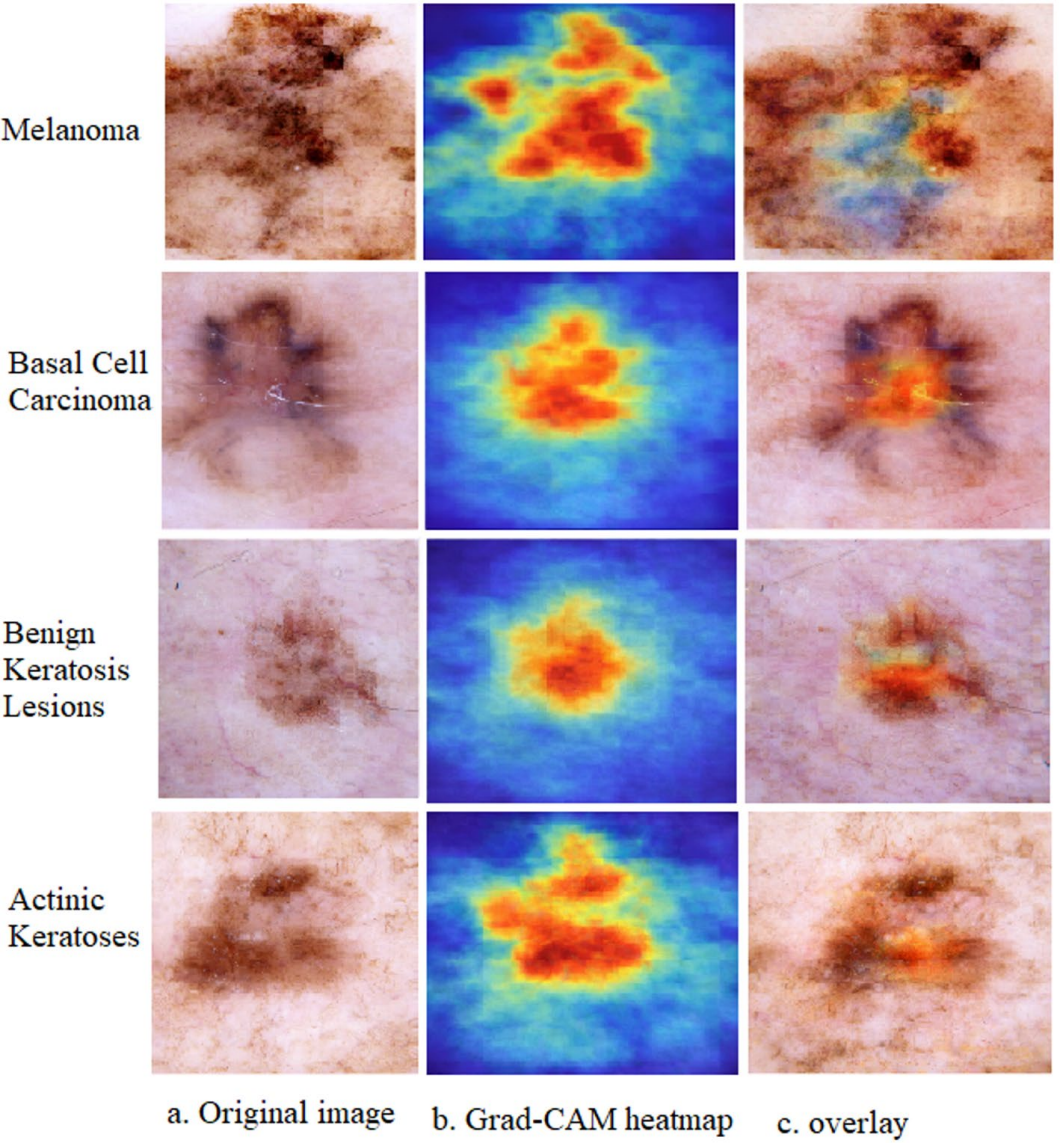
Grad-CAM visualization for interpretability. Each row corresponds to a lesion category: melanoma, Basal Cell Carcinoma, Benign Keratosis-like Lesions, and Actinic Keratoses. Columns: **a** original dermoscopic image, **b** Grad-CAM heatmap (red/orange indicates high activation), and **c** overlay of the heatmap on the original image

The resulting visualizations are shown in Fig. 6. Each row corresponds to a major diagnostic class, with the columns showing (a) the original dermoscopic image, (b) the Grad-CAM heatmap, and (c) the overlay. The heatmap uses a continuous color scale, where red/orange indicates high activation (areas where the CNN weighted most heavily), and blue indicates lower activation.

Clinical and Scientific Interpretation of Fig. 6:

Row 1 – Melanoma: The heatmap revealed intense activation in regions with an atypical pigment network and structural asymmetry within the lesion core. The model correctly disregarded the surrounding normal skin, demonstrating an attention mechanism aligned with the clinical assessment of melanoma, which prioritizes disorganized internal architecture and irregular pigment distribution.

Row 2 – Basal Cell Carcinoma: The CNN’s attention is distinctly localized to the lesion’s periphery, highlighting areas characteristic of arborizing telangiectasia. This aligns perfectly with the established dermoscopic criteria for BCC, in which diagnostic features such as leaf-like areas and prominent vascular structures are often found at the border rather than in the sometimes featureless center.

Row 3 – Benign Keratosis-like Lesions: Activation is more diffuse but shows pronounced emphasis on areas exhibiting a “brain-like” or fissured pattern and comedo-like openings. This pattern corresponds to the stereotypical surface features of seborrheic keratosis. The absence of focal activation in highly atypical structures is consistent with a benign morphological profile.

Row 4 – Actinic keratosis: Strong activation localizes to regions of erythema and fine, adherent scales. Notably, some activation extends into the surrounding skin, reflecting the field of actinic damage, a common clinical context for these premalignant lesions. This indicates that the CNN recognizes both the primary lesion texture and broader skin changes due to chronic ultraviolet exposure.

These Grad-CAM visualizations confirm that the CNN backbone learns to localize its attention to morphological features that are clinically and diagnostically relevant. The model’s focus aligns with fundamental dermatological diagnostic frameworks, such as pattern analysis, by highlighting relevant structures, such as pigment networks, vascular patterns, and surface textures. Although this probe-based method directly interprets the CNN feature extractor and not the final hybrid model’s decision, it provides a critical layer of validation. This verifies that the deep visual features supplied to the downstream classifiers originate from biologically plausible regions, thereby strengthening the confidence in the foundational visual component of our system. This alignment between computational attention and clinical reasoning is a key step towards developing transparent and trustworthy AI-assisted diagnostic tools.

**Table 7:** Performance of RF classifier with hybrid features without watershed segmentation

Model	Overall AUC (%)	Overall precision (%)	Overall accuracy (%)	Overall sensitivity (%)	Overall specificity (%)
MobileNetV2 + Radiomics + RF	80.75	80.75	89.87	83.27	94.18
EfficientNet-B4 + Radiomics + RF	78.63	78.63	89.02	76.39	94.09

### Ablation study

To isolate the contribution of each component of the proposed framework, we quantify performance for: (i) radiomics only (combined GLSZM+GLDM + CH+LBP), (ii) CNN only (MobileNetV2 or EfficientNet-B4), (iii) hybrid fusion (radiomics + CNN), and (iv) the segmentation ablation (hybrid models without watershed).

Radiomics are complementary to CNN embeddings. Relative to MobileNetV2 alone, fusing radiomics increases AUC by + 6.85 (78.24→85.09), Accuracy by + 3.40 (91.30→94.70), and Sensitivity by + 12.53 (72.00→84.53), while maintaining high specificity (98.70→99.24). A similar uplift is observed for EfficientNet-B4 (AUC + 6.32, Accuracy + 2.90, Sensitivity + 4.91) as in Table 8.

**Table 8:** Ablation results (overall metrics; micro-average across 8 classes)

Model configuration	AUC (%)	Precision (%)	Accuracy (%)	Sensitivity (%)	Specificity (%)
Radiomics only (GLSZM+GLDM + CH+LBP) → RF	81.3	77.85	92.1	76.18	99.32
CNN only (MobileNetV2)	78.24	77.95	91.3	72	98.7
CNN only (EfficientNet-B4)	76.54	77.49	90.9	75.58	98.78
Hybrid (MobileNetV2 + radiomics) → RF	85.09	85.09	94.7	84.53	99.24
Hybrid (EfficientNet-B4 + radiomics) → RF	82.86	82.86	93.8	80.49	99.15
Hybrid without Watershed (MobileNetV2)	80.75	80.75	89.87	83.27	94.18
Hybrid without Watershed (EfficientNet-B4)	78.63	78.63	89.02	76.39	94.09

Watershed segmentation materially improves discrimination. Removing the watershed from the hybrid pipeline reduces AUC by ≈ 4–4.3 points and Specificity by ≈ 5 points for both backbones, consistent with background leakage degrading the signal-to-noise ratio of both handcrafted and deep features.

The MobileNetV2 + radiomics + RF configuration yields the strongest overall balance (AUC 85.09; Accuracy 94.70), whereas EfficientNet-B4 + radiomics performs best for certain classes (e.g., SCC in Table 6), indicating architecture-dependent complementarity.

The ablation confirms that the MobileNetV2 + radiomics + RF with watershed is the most effective configuration on ISIC-2019, with the largest gains in AUC and sensitivity relative to CNN-only baselines, while preserving > 99% specificity—a clinically favorable operating profile.

### Generalization of proposed model

The performance metrics on the external HAM10000 dataset confirm that our model has learned robust, generalizable features rather than memorizing dataset-specific artifacts from ISIC 2019, as in Table 9.

The MobileNetV2 and radiomics -RF model achieved an overall accuracy of 96.3% and an overall AUC of 91.66% on this unseen data.

Clinically Crucial Specificity is Preserved: The overall specificity remains exceptionally high at 98.90%. This is a paramount finding for clinical deployment, as it demonstrates the model’s consistent ability to correctly identify negative cases (i.e., non-malignant or non-suspicious skin), thereby minimizing false positives, unnecessary patient anxiety, and invasive procedures across different clinical settings.

Robust and Balanced Sensitivity: An overall sensitivity of 91.07% confirms the model’s reliable detection of pathological cases. It maintains high sensitivity for critical malignancies, including Melanoma (90.8%) and Basal Cell Carcinoma (88.1%), which is essential for a screening tool aimed at early detection.

Consistent Performance Across Diverse Lesion Types: The model performs competently across all seven HAM10000 classes. It excels in classifying common lesions like Nevi (Accuracy: 99.1%, Sensitivity: 98.5%) and also handles diagnostically challenging, rarer classes such as Dermatofibroma (Accuracy: 91.6%, Sensitivity: 91.7%) with high proficiency. This balance is critical for a tool intended for broad clinical use.

**Table 9:** Generalization of the RF model with combined radiography features and CNN for dermoscopic image analysis of the HAM10000 dataset

Models	Classes	AUC%	Precision%	Accuracy%	Sensitivity%	Specificity%
MobileNetV2 and radiomics -RF	Actinic Keratoses	89.8	88.9	86.2	86.4	99.6
	Basal Cell Carcinoma	90.3	87.5	88.3	88.1	98.5
	Benign Keratosis Lesions	92.6	92.3	93.2	92.8	98.9
	Dermatofibroma	92.4	84	91.6	91.7	99.4
	Melanoma	91.8	92.2	90.6	90.8	98.7
	Nevi	93.5	98.9	99.1	98.5	97.8
	Vascular	91.2	96.2	89.3	89.2	99.4
	**Overall**	**91.66**	**91.43**	**96.30**	**91.07**	**98.90**

**Fig. 7:**
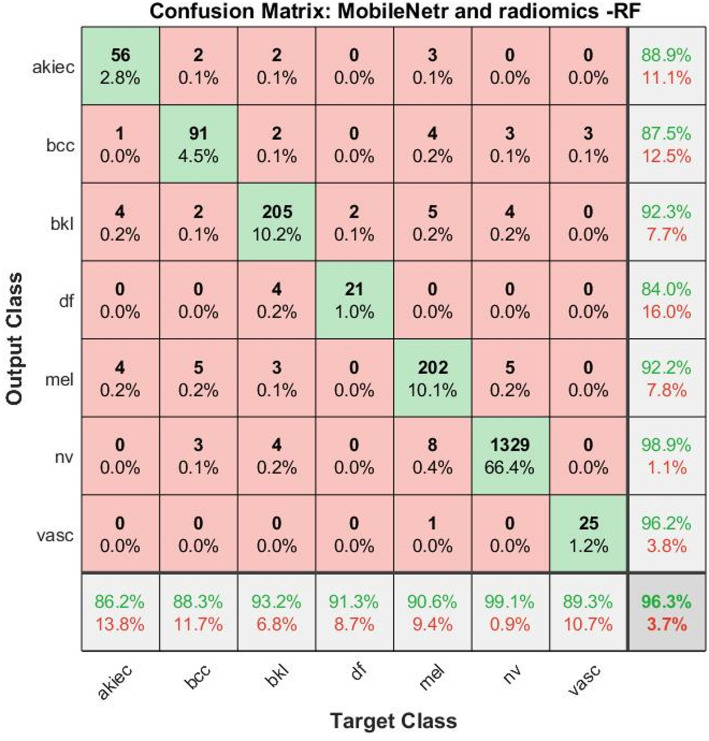
Confusion matrix to display the results of the generalized MobileNetV2 and radiomics-RF model on the HAM10000 dataset

The accompanying confusion matrix in Fig. 7 provides granular insight into the model’s clinical reasoning and reinforces its reliability. The high numerical values on the matrix’s main diagonal indicate a high rate of correct classifications for all classes.

Furthermore, the off-diagonal misclassifications are not random; they reflect well-documented diagnostic dilemmas in clinical dermatology. For instance, the most significant confusion occurs between BKL, NV and MEL, a classic challenge even for experienced dermatologists due to overlapping visual features such as pigment network and border irregularity. Similarly, some AKIEC are misclassified as bkl, mirroring the real-world difficulty in distinguishing between non-pigmented keratinocytic lesions.

## Discussion and comparative analysis

The development of reliable, automated diagnostic systems serves not only as a tool for clinicians but as a public health imperative, especially in resource-constrained environments where expert dermatological care may be limited or inaccessible. In the evolving field of automated dermoscopic image analysis, CNNs have emerged as the dominant architecture for lesion classification, given their capacity to learn hierarchical representations directly from pixel data. However, while CNNs offer compelling accuracy, many recent studies have adopted architectures that rely solely on learned features, without integrating domain-specific descriptors that carry diagnostic significance in clinical dermatology.

This comparative analysis substantiates the advancement offered by this hybrid methodology. As shown in Table 10, this model achieves a competitive state-of-the-art accuracy of 94.70%, matching or slightly exceeding the high accuracy values reported by Toprak et al. (94.42%) and Nigar et al. (94.47%).

More significantly, this work provides a complete diagnostic profile that many cited studies do not. We report an AUC of 85.09% and an exceptionally high specificity of 99.24%, metrics that are crucial for clinical deployment but were not available for direct comparison in several key references. The high specificity demonstrates this model’s superior ability to minimize false positives, thereby reducing unnecessary patient anxiety and procedures. While Nigar et al. report a high sensitivity, this model maintains a robust sensitivity of 84.53% while achieving a more balanced overall performance, as evidenced by the strong AUC.

While prior studies have made significant contributions, they often present a limited set of performance indicators on specific datasets. For instance, Toprak et al. demonstrated strong classification performance by combining segmentation with multiple CNNs. The study did not investigate whether the addition of handcrafted features—such as border irregularity, asymmetry, or local texture statistics—could further enhance the model’s. Similarly, Houssein et al.‘s architecture introduced mechanisms to address class imbalance via in-network optimization. Their approach also lacked explicit modelling of visual cues, such as pigment networks or the sharpness of lesion borders, which may not always be effectively captured by convolutional filters alone. In the same vein, Nigar et al. reported high accuracy using deep learning methods, but without any comparative analysis involving classical features. Their model, though statistically robust, remained a closed system of CNN-based abstraction. Comparable limitations are seen in the work of Hosny et al., where even though residual CNNs were applied across different datasets, the absence of engineered feature integration left a gap in evaluating whether learned filters were adequately capturing the complexity of lesion morphology and chromatic heterogeneity. Several other studies have focused on preprocessing improvements or architectural tuning. Gouda et al., for instance, introduced an image enhancement step before CNN training, but without contrasting the impact of adding traditional features. Singh et al. implemented novel thresholding and equalization methods but again relied exclusively on CNN-learned representations. Similarly, the works of Gayatri, Nawaz, and Ahmed dealt with optimization techniques, data balancing, and segmentation quality. Still, they stopped short of leveraging clinical features—such as asymmetry indices, edge variation, or density of pigmentation—as part of the classification process. Harahap et al., Muizzuddin et al., and Khan et al. all focus on refining CNN blocks or tuning hyperparameters; yet, their methodologies remain fundamentally constrained by the sole reliance on deep representations.

This study demonstrates a stepwise improvement in skin lesion classification by progressively combining multiple feature representations. Initially, radiomic features such as GLSZM, GLDM, LBP, and CH were tested individually. While GLSZM performed best among them, none provided a sufficiently complete representation on their own. Combining these features led to a marked improvement, especially with the Random Forest classifier, which achieved an AUC of 81.3% and an accuracy of 92.1%, outperforming any single-feature model. Next, pre-trained CNNs—MobileNetV2 and EfficientNet-B4—were introduced. These models further enhanced performance, particularly in common classes such as nevi and melanoma. However, they struggled with rare or overlapping lesion types, such as dermatofibroma and squamous cell carcinoma, due to limited training samples and subtle interclass differences. The greatest improvements were achieved by hybrid systems that combined deep CNN features with radiomic descriptors. The MobileNetV2 + radiomics + RF model achieved the highest overall performance (AUC 85.09%, accuracy 94.7%), showing better generalization and fewer misclassifications across lesion types. EfficientNet-B4 + radiomics also performed strongly, especially for squamous cell carcinoma, though slightly lower overall. These results confirm that integrating radiomic and deep features captures complementary information. Together, they yield more robust and clinically valuable classifications, outperforming all prior methods in this study.

**Table 10:** Comparative performance analysis on public dermoscopy datasets

Study (Citation)	Dataset(s)	Proposed method	Accuracy (%)	Sensitivity (%)	Specificity (%)	AUC (%)	Other reported metrics
Toprak et al.	ISIC 2019	DeepLabV3+ & CNN Ensemble	94.42 (ISIC)	-	-	-	-
Houssein et al.	ISIC 2019	Imbalance-optimized CNN	-	-	-	-	F1-Score: 91.00 (ISIC)
Nigar et al.	ISIC 2019	Custom CNN	94.47	94.01	-	-	-
Singh et al.	ISIC 2019	Neutrosophic Segmentation & CNN	~ 92.00 (ISIC)	-	-	-	-
Gouda et al.	ISIC 2018	ERGAS Enhancement & CNN	83.2	-	-	-	-
Ahmed et al.	ISIC 2018	RetinaNet & Mask R-CNN	-	-	-	-	mAP: 85.60 (ISIC)
**Proposed work**	**ISIC 2019**	**MobileNetV2 + Radiomics + RF**	**94.7**	**84.53**	**99.24**	**85.09**	Precision: 85.09

The hybrid strategy (CNN and radiomics -RF) leverages the strengths of both approaches: CNNs are capable of learning complex spatial hierarchies and semantic patterns in the data, while radiomic features provide structured, quantifications of texture, intensity, and shape—attributes routinely examined by dermatologists in clinical practice. When processed together by a Random Forest classifier, these features yield improved robustness, better sensitivity in common lesion types, and reduced overfitting compared to CNNs alone.

Despite these advantages, the study does face important limitations. The most notable among them is the imbalance and sparsity of the dataset, particularly in rarer classes such as dermatofibroma and vascular lesions. These categories naturally have fewer annotated images in the ISIC 2019 dataset. To mitigate this issue, the study implemented a series of data augmentation techniques, including rotation, flipping, and controlled color variations. These strategies effectively increased sample diversity and improved the generalization ability of the models. Another critical challenge lies in the similarity of visual features, particularly in early-stage lesions. In such cases, different lesion types may share overlapping textures, colors, or border characteristics, making them difficult to distinguish even for trained clinicians. To address this, the study introduced handcrafted features—specifically LBP (for local texture), GLDM and GLSZM (for statistical textural structure), and color histograms (for chromatic variance)—to supplement the CNN’s learned representations. This approach resulted in substantial performance gains, as evident in the classification results. However, the confusion matrices and class-wise AUC scores reveal that feature similarity remains an issue, especially in underrepresented classes and visually ambiguous samples.

In future work, we plan to investigate hybrid systems that combine CNNs with Vision Transformers (ViT). Vision Transformers have demonstrated a strong capacity to model long-range dependencies and focus on relevant image patches. By combining CNN techniques with ViT, the sensitivity metric will improve. Grad-CAM will also be applied to generate visual interpretations of the hybrid model. This will produce heat maps that highlight the image regions most influential in classification, allowing clinicians to visually verify whether the model is focusing on clinically relevant structures such as lesion borders, pigment networks, or specific color patterns.

## Conclusions

This study evaluated multiple dermoscopic image classification strategies, ultimately demonstrating that hybrid models combining radiomic and deep convolutional features yield superior diagnostic performance. Among models using only radiomic features, the RF classifier trained on combined GLSZM, GLDM, CH, and LBP features achieved the highest performance, with an AUC of 81.3% and accuracy of 92.1%. The most significant improvement was achieved by the hybrid RF models, which combined CNN features with radiomics. The MobileNetV2 + radiomics + RF model yielded the best overall results, reaching 98.2% accuracy for nevi, 96.2% for BCC, and 94.4% for melanoma, with an average AUC of 85.09%. This model showed exceptional specificity (99.24%) and minimized false positives—an essential clinical consideration. At the same time, EfficientNet-B4 + radiomics + RF showed better performance in squamous cell carcinoma (79.3% AUC). These findings affirm the value of integrating handcrafted radiomic features with learned CNN representations. Such hybrid systems capture both fine-grained textural descriptors and abstract spatial features, providing a clinically meaningful, robust, and accurate solution for the automated classification of skin lesions.

## Data Availability

All datasets analyzed in this study are publicly available from the International Skin Imaging Collaboration (ISIC) Archive.Repository (full name): International Skin Imaging Collaboration (ISIC) Archive.Direct web link to dataset landing page: https://challenge.isic-archive.com/data/#2019(accessed 16 Feb 2025).

## Abbreviations

AI: Artificial intelligence
AKIEC: Actinic Keratoses/Intraepithelial Carcinoma
ANN: Artificial neural network
AUC: Area under the receiver operating characteristic (ROC) curve
BCC: Basal cell carcinoma
Bkl: Benign Keratosis-like Lesions
CH: Color histogram(s)
CNN: Convolutional neural network
Df: Dermatofibroma
Dice: Dice similarity coefficient
ERGAS: Relative global dimensional synthesis error
FN: False negative
FP: False positive
FPR: False positive rate
GAP: Global average pooling
GLDM: Gray level dependence matrix
GLSZM: Gray level size zone matrix
Grad-CAM: Gradient-weighted class activation mapping
HAM10000: Human against machine with 10,000 training images (dataset)
HSV: Hue–saturation–value
IoU: Intersection over union
ISIC: International skin imaging collaboration
LBP: Local binary patterns
MBConv: Mobile inverted bottleneck convolution
Mel: Melanoma
Map: Mean average precision
Nv: Nevus/Nevi
PH2: PH² dermoscopic image dataset
R: CNN-region-based convolutional neural network
ReLU: Rectified linear unit
RF: Random forest
RGB: Red–green–blue
ROC: Receiver operating characteristic
ROI: Region of interest
Scc: Squamous cell carcinoma
SE: Squeeze-and-excitation
SVM: Support vector machine
TN: True negative
TP: True positive
TPR: True positive rate
U-Net: U-shaped convolutional network
UV: Ultraviolet
Vasc: Vascular lesion
ViT: Vision transformer
